# How Should We Communicate Information Regarding Birth Choices to Women?: An Online Randomised Survey

**DOI:** 10.1111/1471-0528.18314

**Published:** 2025-07-28

**Authors:** Victoria Bradley, Alexandra Hunt, Eve Bunni, Carol Kingdon, Leila Finikarides, Laura Bonnett, Caroline Cunningham, Christy Burden, Jo Ficquet, William Parry‐Smith, Elena Oteroromero, Arezou Rezvani, Mairead Black, Gemma Clayton, Gordon C. S. Smith, Deborah Lawlor, Jon Heron, Abi Merriel, the Options Study Collaborative Group, Deborah Lawlor, Gordon Smith, Jane Norman, Jon Heron, Louise Kenny, Sheelagh McGuinness, Anna Davies, Dame Tina Lavender, Christy Burden, Jonathan Ives, Simon Grant, Sherif Abdel‐Fattah, Danya Bakhbakhi, Laura Bonnet, Andrew Demetri, Mairead Black, Sam Finnikin, Amie Wilson, Alexandra Freeman, Katherine Birchenall, Louise Kenny, Joanne Johnson, Jane Norman

**Affiliations:** ^1^ Department of Women's and Children's Health, Institute of Life Course and Medical Sciences University of Liverpool Liverpool UK; ^2^ Department of Health Data Science University of Liverpool Liverpool UK; ^3^ Institute of Life Course and Medical Sciences, Department of Women's and Children's Health, Centre for Women's Health Research University of Liverpool Liverpool UK; ^4^ Former Winton Centre for Risk and Evidence Communication University of Cambridge Cambridge UK; ^5^ Liverpool Women's NHS Foundation Trust Liverpool UK; ^6^ Bristol Medical School The University of Bristol Bristol UK; ^7^ Royal United Hospitals Bath Bath UK; ^8^ University of Keele Newcastle UK; ^9^ Cambridge University Hospitals NHS Foundation Trust Cambridge UK; ^10^ University College London Hospitals NHS Foundation Trust London UK; ^11^ Aberdeen Centre for Women's Health Research University of Aberdeen Aberdeen UK; ^12^ Department of Obstetrics & Gynaecology, Department of Obstetrics and Gynaecology University of Cambridge Cambridge UK

**Keywords:** birth, decision making, risk communication

## Abstract

**Objective:**

To identify which risk communication graphics are easiest to understand when communicating risks surrounding birth, and investigate the effect of numeracy and health literacy on understanding.

**Design:**

Online survey with randomised content.

**Setting:**

UK.

**Population:**

Women and their partners, healthcare professionals and stakeholders in the UK.

**Methods:**

Seven candidate graphics (‘10‐person’, ‘100‐person’, ‘10‐circle’, ‘100‐circle’ icon arrays, ‘bar charts’, ‘pie charts’ and ‘words’) were co‐produced with a patient and public involvement group. These were used to develop seven online surveys. Participants were recruited using social media and adverts in participating hospitals. Each participant was randomised to see one of seven surveys, the survey questions remained the same, the graphics varied. Data were collected on risk perception, perceived ease of understanding and preference, alongside demographic factors, numeracy skills and health literacy.

**Main Outcome Measures:**

Objective comprehension and subjective preference for graphics.

**Results:**

There were 858 participants, 771 women/partners/parents and 87 healthcare professionals and stakeholders. 70% of participants answered all four numeracy questions correctly, and respondents reported high health literacy (76.5% can understand health material, 72.8% were comfortable completing medical forms). All graphics were understood; however, the ‘100‐person’ icon array elicited the best score when comparing two risks (mean score 97.5% compared to next best performing graphic 95.8%), 41% believed it was the easiest to understand and 36% selected it as the preferred graphic.

**Conclusions:**

All graphics are well understood. The ‘100‐person’ icon array scored best in terms of risk comparison and participant preference. Using this graphic may support high‐quality communication of risk in maternity care.

## 1 Introduction

High‐quality communication and information is vital to enable women to make informed decisions about their birth [1, 2, 3]. The way women are giving birth is changing; 42 in 100 women experienced a caesarean birth in 2023/24 [4] compared to below 30 in 100 women between 2018/19 [5] with around 23% of these women being over 35 years old [4]. Often, women report that they are not informed of the risks and benefits of vaginal birth, and the risks surrounding caesarean and instrumental birth are not communicated effectively [6, 7]. The resultant lack of feeling in control may contribute to poor birth experiences, birth trauma [7] and post‐traumatic stress disorder [6, 7, 8, 9]. Improving the way information about birth, including risks and benefits of different modes of birth, and improving birth preparedness through antenatal education [10] may improve birth experiences [7].

When choosing mode of birth, women gain information from a variety of healthcare professionals including doctors and midwives, lived experiences, word of mouth, family and friends, and social media [8]. Staff are poorly prepared for effective shared decision making, risk communication, and lack the tools and time to support these discussions [9]. Women value information from healthcare professionals [8], creating an opportunity to provide high‐quality, accessible, and evidence‐based information. Shared decision making is a term used by organisations such as NHS England, referring to the discussion and weighing up of risks and benefits of each mode of birth in a patient centred way [11]. However, in maternity care there is a move towards terms such as ‘supported decision making’ to reflect patient autonomy and empowering decision making while providing accessible information alongside adjuncts (e.g. graphics) to aid understanding [11, 12, 13, 14]. Providing graphics alongside numerical risks improves understanding and patient satisfaction [15, 16]. Risk communication may also play an important role in aiding the comparison of two risks [17, 18, 19].

Studies have examined graphics in the context of survival, death, and the level of worry the risk causes [20]. However, few studies have investigated the presentation of risks in maternity care, despite its unique nature [20]. It is unique due to the physiological changes of pregnancy, the opportunity for risks to evolve rapidly, and the maternal‐fetal dyad where risks and benefits can impact on the mother or fetus differently [20]. Risk communication research is largely limited to antenatal trisomy screening [21] and few studies examining the most suitable graphics [20]. However, the suitability of these graphics for those with lower health literacy and numeracy abilities has been investigated less.

Numeracy and literacy ability varies among populations, and risks can be easily misunderstood [22]. Yet, many risks presented to patients rely on having a degree of understanding of percentages, proportions, and fractions. Lower health literacy may be associated with a lack of understanding of test results, negative beliefs surrounding medicine, and reduced understanding of how pregnancy impacts the health of the mother and fetus [23, 24]. Lower levels of education and high levels of unemployment are high deprivation characteristics associated with poorer health outcomes [25, 26]. Therefore, ensuring patients from all backgrounds can easily understand information, regardless of their numeracy or literacy ability, is important.

This study aims to develop candidate risk communication graphics, identify which are easiest to understand when communicating risks surrounding birth, and investigate the effect of numeracy and health literacy on understanding.

## 2 Methods

These methods are divided into two parts: the development of the graphics and the online survey.

### 2.1 Co‐Producing the Risk Communication Graphic

Candidate risk communication graphics were co‐produced [27] with parents and professionals in a series of patient and public involvement (PPI) groups. This method allows stakeholders to collaborate with researchers throughout an entire project; holding value in lived experiences, equal value being placed on the contributions from all members and having shared ownership of outputs [27, 28, 29, 30, 31]. To contextualise the purpose of the risk communication graphics, an existing set of information about vaginal birth [32] was discussed. With this context in mind, the PPI group discussed a variety of ways to present these risks and generated ideas, multiple versions of which were collaboratively produced alongside a graphic designer. Iterative input was sought until there was a consensus on the format of the candidate graphics. The PPI group narrowed down the number of graphics to be taken forward into the survey.

### 2.2 Risk Communication Online Survey

The survey has been designed and reported according to CHERRIES guidelines [33] and approved by Greater Manchester Research Ethics Committee (23/NW/0370).

### 2.3 Survey Development

An online survey to examine the candidate risk communication graphics and the impact of numeracy and literacy skills on the graphic was produced. It was developed and extensively tested for functionality and usability in two in‐person PPI groups and within the study team. In‐person PPI groups were held in a community children's centre and at the lead hospital site. Both venues were located within areas of high deprivation [25].

After the survey was developed, seven separate versions, identical except for candidate graphics, were created (File S1).

### 2.4 Study Design

After receiving informed consent via REDCap, each participant was randomised to one of the seven surveys, stratified according to their parent or professional status. They could choose to be entered into a prize draw for one of four £25 vouchers.

We collected data on participant demographics, numeracy, and health literacy in addition to their response to the seven candidate graphics.

To assess numeracy ability, we used the Schwartz numeracy test [22] (three questions) and a question from the expanded numeracy test by Lipkus et al. [34] to assess the participants' understanding of proportions. This was considered important as many risks are reported as proportions. Questions included: which of four proportions represented the highest chance of something happening; how many times the coin would land on heads if flipped 1000 times; identifying the proportion which matches 1% of 1000 people who win a scratch card lottery; and converting a proportion representing the chance of winning a raffle prize into a percentage of people who would win the prize. This approach was chosen [22, 34] rather than the Berlin numeracy test [35] as PPI members were not comfortable with its complexity and believed it to be unsuitable for an audience who may have a poorer numeracy ability.

Health literacy was assessed using the two parts of the single item literacy screening questionnaire [36]. The first examined how often the participants needed help with health‐related materials [36]. The second examined how comfortable participants were filling out health‐related forms [36]. The single item literacy screening questionnaire [36] was used due to its rapid nature.

Both the health literacy and numeracy sections were presented as multiple choice questions with an additional “Don't know” option for the numeracy questions, in line with PPI input and CHERRIES guidelines [33].

Each survey arm contained a single type of graphic with a variety of numbers for each question to assess the suitability of different types of graphics to communicate different numbers. Each graphic was accompanied by a question asking how surprised the participant would be if this event happened to them. This was designed to gauge the participants' understanding of what the graphic and number are communicating about the likelihood of future events [37]. We also asked participants to look at two of the same graphic presenting different risks. We then asked them which one represented the biggest number as the ability to distinguish between two risks is essential when comparing different birthing methods.

### 2.5 Population

To participate in the survey, participants needed to have access to a device with an internet connection. They had to: be at least 18 years old, understand English, be able to consent, be pregnant, or have previously given birth, have a partner who has given birth, or a healthcare professional involved in discussions around mode of birth or a relevant stakeholder for example, from a birth charity.

### 2.6 Recruitment

This survey was online, hosted on REDCap [38, 39] which is a website used to collect and manage data for studies. This was open between May and June 2024. Participants were recruited via social media channels including Facebook, Instagram and X as well as via posters in participating hospital sites. The survey was also shared via posters with children's centres located within deprived areas. Recruitment materials and social media impressions are available in File S1.

### 2.7 Data Analysis

Completeness checks were carried out prior to analysis. Partially completed responses were included within the analysis, with the denominator altered accordingly.

Data analysis was carried out using R (4.4.1.) [40]. Descriptive statistics and visualisation were used to assess characteristics of the collected data including frequencies, interquartile ranges, and modes.

## 3 Results

### 3.1 Co‐Producing the Risk Communication Tools

Seven PPI groups were held involving midwives (3), parents (12), researchers (5), members from interested organisations (2) and a statistician, and additional meetings with risk communication experts (2). The PPI group developed seven candidate graphics to be tested within the online survey (File S2: Figure S1).

### 3.2 Risk Communication Online Survey

1044 participants responded to the online survey. 186 participants provided consent but did not answer any questions and were excluded. A total of 858 responses were included in the analysis, 824 (96%) of which were complete responses. These respondents were randomised to one of seven surveys, with numbers of participants randomised to each survey ranging from 101 to 136 (File S2: Figure S2).

### 3.3 Demographics (File S2: Table S1)

Of the 858 respondents, 87 were professionals (10%) and 771 were parents (90%). Most were aged between 31 and 40 (69%, 596) and female (97%, 830). Most participants were of White British ethnicity (82%, 704) and had a university education (86%, 738). The majority of participants were either on maternity leave (36%, 306) or in full time employment (33%, 287) and the participants were distributed across the United Kingdom. Most parents had either given birth in the past (65%, 502/771) or were currently pregnant (26%, 200/771) and most professionals were midwives (53%, 46/87) and doctors (24%, 21/87). The characteristics of respondents answering each survey were similar in age, education status and parent characteristics for example, number of children (File S2: Table S2). Participants within each survey were mostly White British, however 100% of participants completing survey 3 (‘100‐circle icon array’) were White British (File S2: Table S2). All other surveys had participants who were not White British.

### 3.4 Numeracy

All 858 participants completed all four numeracy questions (Table 1). Participants generally scored highly in the numeracy questions. The professionals generally scored higher, with 75% of professionals choosing the correct answer on all four questions compared with 70% of parents.

**TABLE 1:** Numeracy and health literacy questions and the percentage of participants who answered them correctly.

Proportion of correct numeracy answers (%)	Professional	Parent	Total
**Numeracy questions**			
Highest proportion	84/87 (97%)	737/771 (96%)	821/858 (96%)
Coin flip	81/87 (93%)	723/771 (94%)	804/858 (94%)
Scratch card	75/87 (86%)	645/771 (84%)	720/858 (84%)
Lottery	72/87 (83%)	599/771 (78%)	671/858 (78%)
**Health literacy questions**			
How often do you need help with health‐related materials?			
Never	62/87 (71.3%)	594/771 (77%)	656/858 (76.5%)
Rarely	13/87 (14.9%)	95/771 (12.3%)	108/858 (12.6%)
Sometimes	9/87 (10.3%)	53/771 (6.9%)	62/858 (7.2%)
Often	2/87 (2.3%)	17/771 (2.2%)	19/858 (2.2%)
Always	1/87 (1.1%)	12/771 (1.6%)	13/858 (1.5%)
How comfortable you are with filling out health‐related forms?			
Not at all	0	7/771 (0.9%)	7/858 (0.8%)
A little bit	2/87 (2.3%)	18/771 (2.3%)	20/858 (2.3%)
Somewhat	4/87 (4.6%)	43/771 (5.6%)	47/858 (5.5%)
Quite a bit	20/87 (23%)	139/771 (18.0%)	159/858 (18.5%)
Extremely	61/87 (70.1%)	564/771 (73.2%)	625/858 (72.8%)

### 3.5 Health Literacy

Two questions examined the health literacy of the participants. All 858 participants answered both questions (Table 1). Most participants reported that they never need help when reading health‐related information (77% (656/858), parents 77% (594/771), professionals 71% (62/87)) and that they are extremely comfortable filling out medical forms (73% (625/858), parents 73% (564/771), professionals 70% (61/87)). (File S2: Figure S3).

#### 3.5.1 Risk Communication (Figure 1A, File S2: Table S3)

**FIGURE 1:**
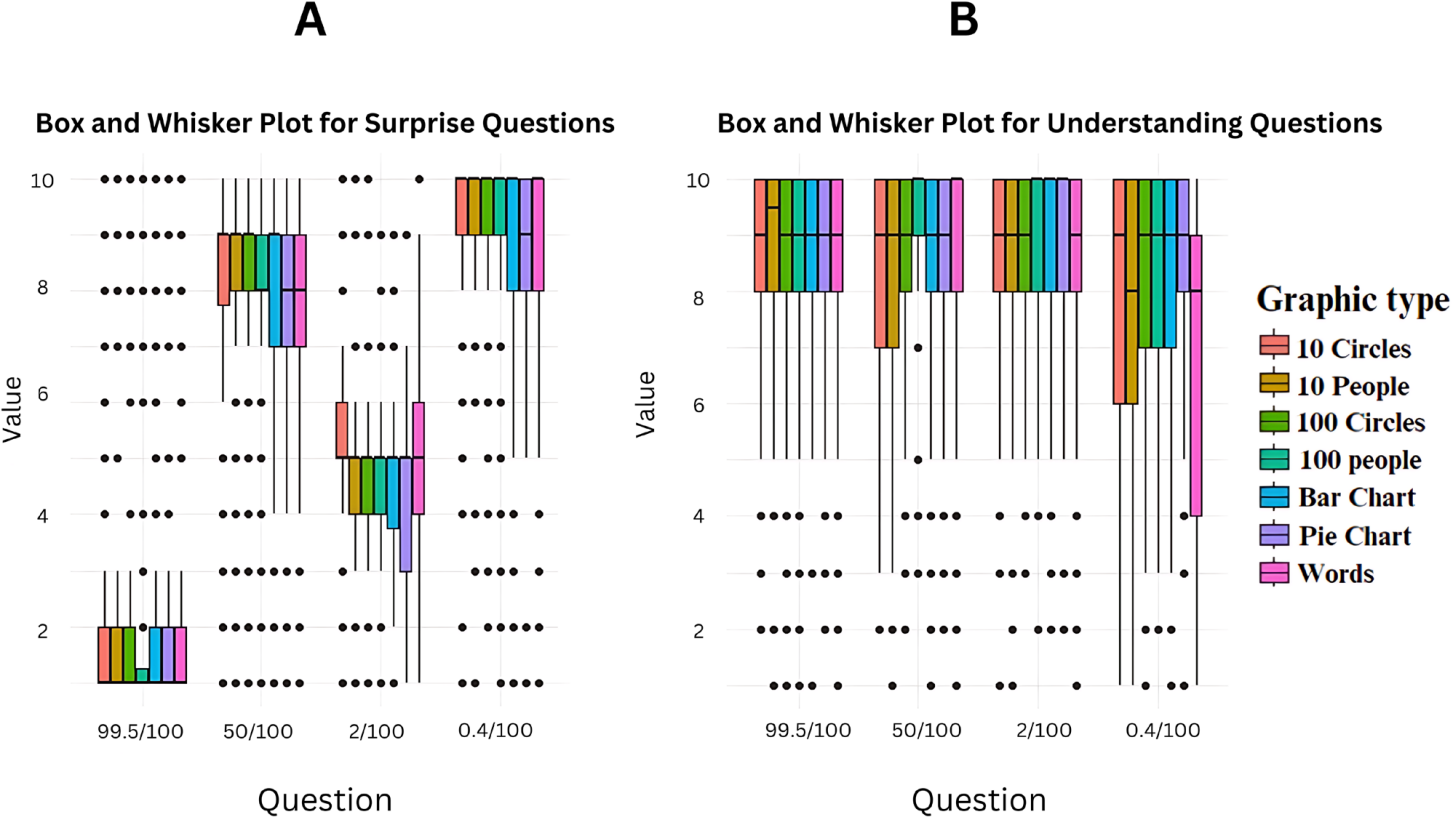
(A) Box and whisker plots displaying the level of surprise for each graphic type and related risk question. One represents not at all surprised and ten represents extremely surprised. (B) Box and whisker plots displaying the reported ease of understanding for each graphic type and related risk question. One represents the hardest to understand and ten represents the easiest to understand.

The results in Figure 1A show that the median estimates of the magnitude of risks are similar between graphics. The exception seemed to be for the risk “99.5/100”, where ‘100‐people’ showed the lowest level of surprise with the same median of 1 (IQR 1–1.25) but an IQR of 1–2 for all other graphic types (Figure 1A). For the smallest risks, 0.4% and 2%, ‘bar charts’, ‘pie charts’ and ‘words’ had higher medians and a larger IQR than the 10 and 100 circles/people icon arrays. With the exception of 99.5/100, the self‐reported surprise with ‘words’ had the widest IQR.

#### 3.5.2 Subjective Understanding (Figure 1B, File S2: Table S3)

The reported level of understanding was the same across all seven risk communication tools for 99.5/100 and 50/100. However, the reported level of understanding for the graphics depicting a risk of 2/100 and 0.4/100 showed some variation in medians and IQRs. For 2/100 the graphic with the highest median and smallest IQR was the ‘100‐person’ icon array (median 10, IQR 9–10). The ‘10‐circle’ (median 9 IQR 7–10) and ‘10‐person’ graphics (median 9, IQR 7–10) elicited larger IQRs. For 0.4/100, ‘words' had the lowest median and largest IQR (median 8, IQR 4–9) whereas the ‘pie charts' elicited a higher median and smaller IQR (median 9, IQR 8–10).

#### 3.5.3 Comparing Two Risks (Figure 2, Table S4)

**FIGURE 2:**
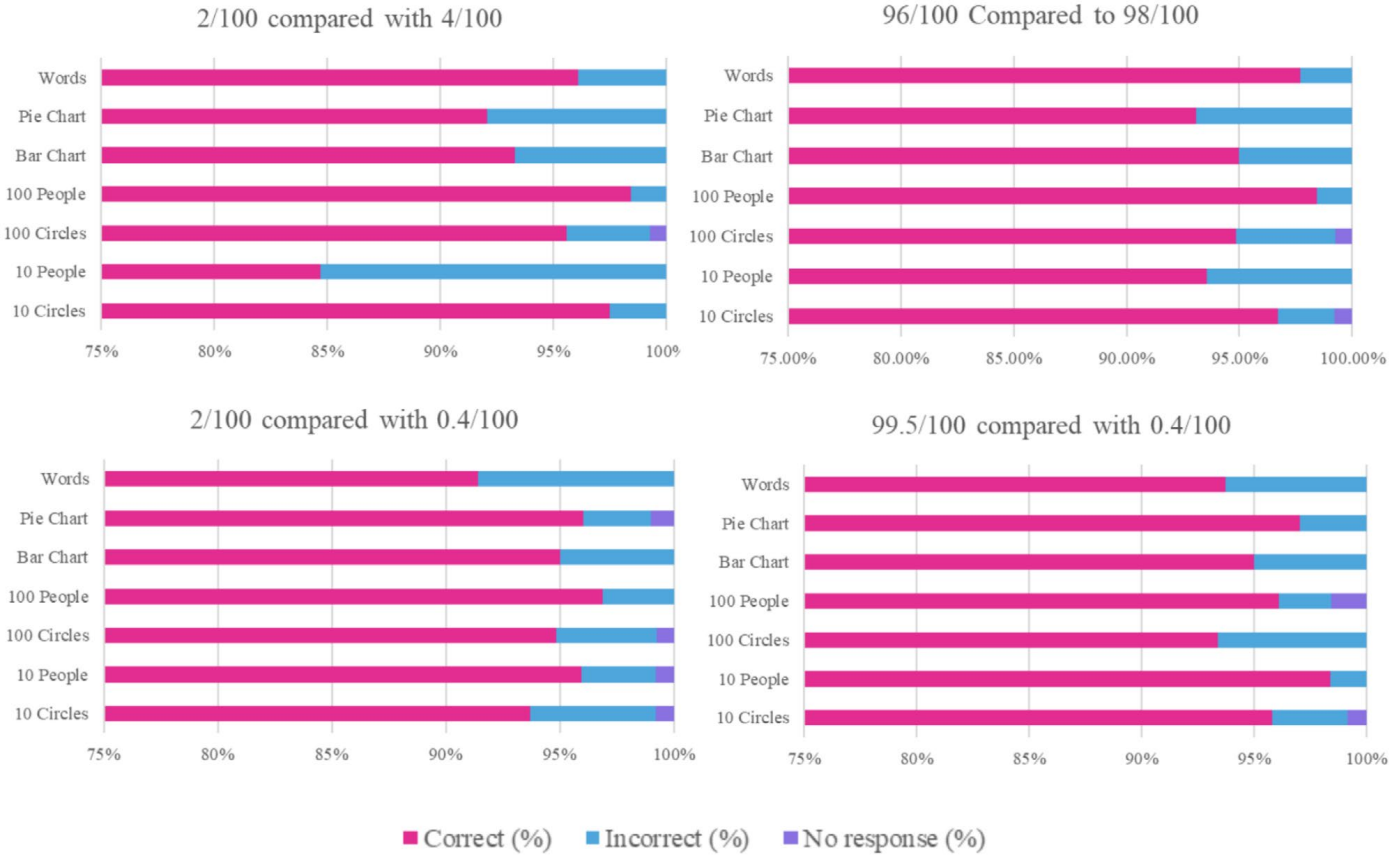
Stacked bar graphs showing the percentage of participants correctly identifying the largest risk for each graphic type.

Participants were shown two risks using the same graphic but different numbers and asked to identify which represented the bigger risk. Participants answered four questions comparing 2/100 and 4/100, 96/100 and 98/100, 2/100 and 0.4/100 and, 99.5/100 and 0.4/100. The ability to identify the largest risk was similar across all graphic types, however there were small variations. When comparing 2/100 and 4/100, participants who saw the ‘10‐person’ icon array got the question wrong most frequently (15%) compared to the ‘100‐person’ icon array where 2% answered incorrectly. The ‘100‐person’ icon array seemed to perform best when comparing two risks for three of the comparisons (98.5%, 98.5%, 96.9%). The only instance where ‘100‐person’ icon array did not elicit the greatest percentage of correct answers was when comparing 99.5/100 and 0.4/100 where it performed third best (96.1%) after the ‘10‐person’ icon array (98.4%) and the ‘pie charts’ (97.0%).

### 3.6 Subjective Preference of Graphic

When participants were shown all seven graphics, the ‘100‐person’ icon array was preferred (36%, 309/858) although it is not possible to say whether this preference occurred by chance. A greater proportion of professionals ranked the ‘100‐person’ icon array as their preferred graphic (45%, 39/87) compared with parents (35%, 270/771). This was closely followed by ‘words’ (18%) (155/858) and the ‘100‐circle’ icon array (16%, 138/858). The ‘100‐person’ icon array was ranked the easiest to understand by the most people (41%, 353/858), with 48.3% (42/87) of professionals and 40% (311/771) of parents ranking it highest.

#### 3.6.1 Numeracy, Health Literacy and Preferred Graphic (Figure 3)

**FIGURE 3:**
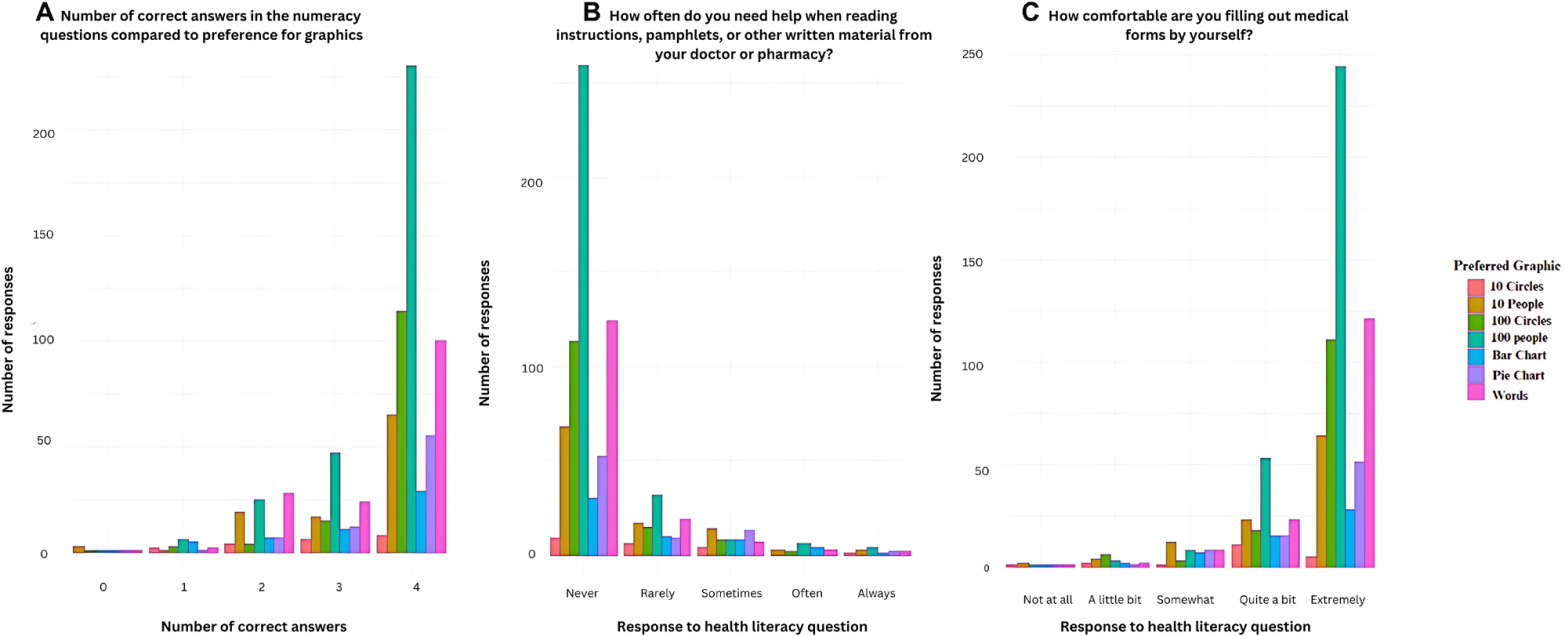
(A) Perfomance in numeracy questions compared to the reported preferred graphic (B) Single item literacy screening question one scores compared with preferred graphic. (C) Single item literacy screening question two compared with preferred graphics.

Using graphical representations, we observed patterns and potential relationships between reported health literacy and preferred graphics. Most groups have a preference of the ‘100‐person’ icon array, regardless of these other factors (File S2: Figure S4). However, those who did not get any numeracy questions correct, or two questions correct preferred the ‘10‐person’ icon array but their performance when comparing two risks was poorer than with the ‘100‐person’ icon array. Whereas those who got all four numeracy questions correct, preferred the ‘100‐person’ icon array and performed better when comparing two risks.

When comparing individual's performance during the risk questions and surprise questions, there is no particular graphic which performs best (File S2: Figure S5). This is also the case when comparing participants' ease interpreting a graphic and their performance during the risk questions (File S2: Figure S6).

When comparing health literacy to the number of correctly answered risk questions, the graphic used did not seem to make a difference for those who are most confident with their health literacy. However, those who are less literate may perform better when using the ‘100‐person’ icon array or ‘10‐circle’ icon array (File S2: Figure S7).

## 4 Discussion

### 4.1 Main Findings

This study aimed to develop candidate risk communication graphics and identify which are most effective for communicating risks surrounding birth, and explore the effect of numeracy and health literacy on understanding. Most participants rated themselves as comfortable with health‐related information and being confident with filling out medical forms and performed strongly in the numeracy questions.

The ‘100‐person’ icon array was voted as the preferred graphic, scoring higher in the subjective comprehension questions and eliciting the highest scores when comparing two risks in three out of four questions. However, the performance across all graphics was good and the ‘100‐person’ icon array was only minimally preferred compared to other graphics and therefore, other graphic types may be suitable when communicating risks. Because of the lack of low scores on the numeracy and health literacy questions we were unable to perform a moderation analysis.

### 4.2 Strengths & Limitations

The multi‐prong recruitment strategy using social media, emails and posters in participating sites was a strength yielding a high‐response rate. The use of social media recruitment can be beneficial to the inclusion of underserved communities [20] and encourage a wider audience to engage in research. Despite the strong social media recruitment and presence in local children's centres, the majority of respondents were not from underserved communities. Nonetheless, we did achieve some reach. Individuals who do not understand English could not participate, but it is unclear to what extent this impacted recruitment, though we know people with English as a second language did participate.

We included both parents and professionals in our large sample. These groups had similar preferences of graphics and performance within the numeracy questions and reported health literacy. There were 87 healthcare professionals in total, meaning there were fewer professionals in each group.

The examination of health literacy relied on the Single Item Literacy Screening questionnaire [36] in a multiple choice format. The multiple choice format relies on participants to self‐report their health literacy, reducing its reliability. There are other options to examine health literacy such as the short assessment of health literacy in English [41], involving 18 items, the test of functional health literacy which involves two parts and the Newest Vital Sign assessment of health literacy [42]. However, these tests are longer, not appropriate for an online survey, and/or may deter participants from completing the questions. We sought PPI views on these validated long and short questions before finalising the questionnaire design. We changed the questionnaire to the single item screening questionnaire based on PPI feedback. We consider this a strength which positively contributed to our high‐response rate.

The examination of numeracy involved multiple choice questions which may have increased the number of correct answers and ease of answering compared to other numeracy tests. The multiple choice format and questions were used due to PPI input regarding the more challenging nature of the Berlin [35] numeracy test. PPI groups felt this format was more suitable for people with lower numeracy or literacy skills, aiming to prevent the deterrence of participants. However, reducing the difficulty of questions may have also increased the number of correct answers and decreased our ability to assess participants with lower numeracy skills.

The number of participants with a university education and of White British ethnicity was high within our population, but not dissimilar to the general birthing population. The study team made efforts to recruit from diverse groups in deprived areas. We did not achieve the reach we sought with individuals of lower educational backgrounds, which is a common limitation of general surveys.

### 4.3 Interpretation

This study found the preferred and most often correctly interpreted graphic type was the ‘100‐person’ icon array followed by the ‘100‐circle’ icon array and ‘words’. This finding is similar to a study by Ferguson et al. which found that figures were preferred over circles when used in icon arrays [20] when examining chance of survival and death. Whilst, their study compares figures and circles, it did not examine the preference in number of figures or circles presented. Our study found there was a difference in preference and performance dependent on the number of circles or figures within a graphic which also impacted their perceived ease of understanding.

Ferguson et al. had a similar population to the participants within this study [20]. Unlike our study, Ferguson et al. found the preference within their study to be ‘pie charts’. We found understanding between two risks with ‘pie charts’ did not elicit a larger percentage of correct answers or votes for being easiest to understand compared to other graphic formats. Ferguson et al. [20] also compared participants understanding between graphics and found participants understanding to be generally good. However, they compared two different numerical risks using two different types of graphics. In contrast, we examined understanding between two risks using the same type of graphic. We found that most participants were able to correctly identify the largest risk when presented with two risks using the same graphic type.

Garcia‐Retamero et al. found that adding icon arrays eliminated framing bias [43]. These icon arrays also had the greatest benefit to young adults with low numeracy [44]. Whilst our study had a small population of people with low numeracy, we found the ‘100‐person’ icon array to elicit the greatest number of correct responses, receive the most votes for ease of understanding and therefore, may it be useful for decision making. They also found that involving the audience of interest in the design, assessment and sharing of the graphics to be vital [44]. Our study ensured consistent PPI involvement to maximise the suitability of graphics.

Oudhoff and Timmermans [45] found that a lower numeracy ability was associated with rating perceived likelihood lower, and a high numeracy ability was not associated with any effect on the perceived likelihood of a risk. Despite having a large sample size, our group with a lower numeracy ability was still small. Therefore, performing statistical analysis to compare the numeracy ability and understanding of various graphics was not appropriate.

It was beyond the scope of this study to assess risk communication in relation to mode of birth. Risk communication regarding mode of birth requires improvement. There are many tools available for communicating information regarding mode of birth, but there is little standardised information. Further work improving the graphics to effectively communicate uncertainty within risks is empirical due to the nature of risks within maternity care [46] and the quality of the evidence. Further research should include participants with lower numeracy skills and education.

## 5 Conclusions

Risk communication is vital to enable women to make decisions about birth. Tools which enable comparison of risks may be particularly important for aiding the decision making between different birthing methods. We saw little impact of numeracy and literacy skills on the interpretation of risk, however, our participants were not sufficiently varied in numeracy and literacy skills to identify variation in risk interpretation as a result of these characteristics. Whilst all graphics performed well, we found the ‘100‐person icon array’ seemed to perform best in terms of empirical understanding, ease of interpretation and preference of both women and health professionals.

## Author Contributions

A.M. and V.B. designed the survey alongside P.P.I. input. A.M., E.B., V.B., and C.K. aided the acquisition of data. A.M., A.H., E.B., and V.B. analysed and interpreted the data. V.B., A.H., and A.M. drafted the first version of this manuscript. V.B., A.H., E.B., C.K., A.F., L.F., L.B., C.C., C.B., J.F., W.P.‐S., E.O., A.R., M.B., G.C., M.L., L.K., J.N., G.S., D.L., J.H., and A.M. revised the article. Options Collaborative group members contributed to the study design and approved the final version of this manuscript.

## Ethics Statement

This work has been approved by the Greater Manchester REC (23/NW/0370). This was approved on the 3rd of January 2024.

## Conflicts of Interest

The authors declare no conflicts of interest.

## Supporting information


**Data S1:** bjo18314_sup‐0001‐Appendix 1.docx.

**Data S2:** bjo18314_sup‐0002‐Supplementary‐Figures‐Tables.docx.

## Data Availability

The data that support the findings of this study are available from the corresponding author upon reasonable request.

## References

[1] Judgment: Montgomery (Appellant) v Lanarkshire Health Board (Respondent) (Scotland) (2013), https://www.supremecourt.uk/cases/docs/uksc‐2013‐0136‐judgment.pdf.

[2] S. W. Chan , E. Tulloch , E. S. Cooper , A. Smith , W. Wojcik , and J. E. Norman , “Montgomery and Informed Consent: Where Are We Now?,” BMJ (Clinical Research Ed.) 357, no. 1 (2017): j2224. 10.1136/bmj.j2224 28500035

[3] Montgomery and Informed Consent 2024, https://www.themdu.com/guidance‐and‐advice/guides/montgomery‐and‐informed‐consent.

[4] NHS England , NHS Maternity Statistics, England, 2023–24 (2024), https://digital.nhs.uk/data‐and‐information/publications/statistical/nhs‐maternity‐statistics/2023‐24.

[5] NHS Digital , NHS Maternity Statistics, England 2018–19, https://digital.nhs.uk/data‐and‐information/publications/statistical/nhs‐maternity‐statistics/2018‐19.

[6] Dignity in Childbirth , Birthrights, https://www.birthrights.org.uk/campaigns‐research/dignity‐in‐childbirth/.

[7] K. Thomas , “Listen to Mums: Ending the Postcode Lottery on Perinatal Care,” (2024), https://www.theo‐clarke.org.uk/sites/www.theo‐clarke.org.uk/files/2024‐05/Birth%20Trauma%20Inquiry%20Report%20for%20Publication_May13_2024.pdf.

[8] C. Yuill , C. McCourt , H. Cheyne , and N. Leister , “Women's Experiences of Decision‐Making and Informed Choice About Pregnancy and Birth Care: A Systematic Review and Meta‐Synthesis of Qualitative Research,” BMC Pregnancy and Childbirth 20, no. 1 (2020): 1–21. 10.1186/s12884-020-03023-6 PMC7285707 32517734

[9] K. Hardman , A. Davies , A. Demetri , et al., “Maternity Healthcare Professionals' Experiences of Supporting Women in Decision‐Making for Labour and Birth: A Qualitative Study,” BMJ Open 14 (2024): e080961. 10.1136/bmjopen-2023-080961 PMC11057275 38684269

[10] A. Merriel , M. Toolan , M. Lynch , et al., “Codesign and Refinement of an Optimised Antenatal Education Session to Better Inform Women and Prepare Them for Labour and Birth,” BMJ Open Quality 13, no. 2 (2024): e002731. 10.1136/bmjoq-2023-002731 PMC11168157 38858078

[11] “Informed Consent and Shared Decision Making in Obstetrics and Gynecology: ACOG Committee Opinion, Number 819,” Obstetrics and Gynecology 137, no. 2 (2021): e34–e41. 33481530 10.1097/AOG.0000000000004247

[12] The Royal College of Midwives , “Informed Decision Making,” (2022).

[13] E. White , A. Davies , A. Demetri , et al., “Women's Perspectives of Decision‐Making for Labour and Birth: A Qualitative Antenatal‐Postnatal Paired Interview Study,” BMJ Open 15, no. 6 (2025): e096171. 10.1136/bmjopen-2024-096171 PMC12142090 40467318

[14] L. Murugesu , O. C. Damman , D. R. M. Timmermans , et al., “Health Literate‐Sensitive Shared Decision‐Making in Maternity Care: Needs for Support Among Maternity Care Professionals in The Netherlands,” BMC Pregnancy and Childbirth 23, no. 1 (2023): 594. 37605153 10.1186/s12884-023-05915-9 PMC10440871

[15] A. L. J. Freeman , “How to Communicate Evidence to Patients,” Drug and Therapeutics Bulletin 57, no. 8 (2019): 119–124. 31345957 10.1136/dtb.2019.000008 PMC6678057

[16] D. Zipkin , C. A. Umscheid , N. L. Keating , et al., “Evidence‐Based Risk Communication: A Systematic Review,” Annals of Internal Medicine 161, no. 4 (2014): 270–280. 25133362 10.7326/M14-0295

[17] Understanding Risk , “BMJ Best Practice,” https://bestpractice.bmj.com/info/us/toolkit/practise‐ebm/understanding‐risk/.

[18] T. Rakow , E. Blackshaw , C. Pagel , and D. S. Spiegelhalter , “Comparing What to What, on What Scale? The Impact of Item Comparisons and Reference Points in Communicating Risk and Uncertainty,” Journal of Behavioral Decision Making 31, no. 4 (2018): 547–561.

[19] D. Spiegelhalter , “Risk and Uncertainty Communication,” Annual Review of Statistics and Its Application 4, no. 1 (2017): 31–60.

[20] M. Ferguson , G. D. Shapiro , and S. D. McDonald , “Understanding and Preferences Regarding Risk Communication During Pregnancy: A Survey to Facilitate Provider Communication With Patients,” American Journal of Obstetrics & Gynecology MFM 5, no. 6 (2023): 100929. 36931434 10.1016/j.ajogmf.2023.100929

[21] L. M. Hunt , H. Castaneda , and K. B. De Voogd , “Do Notions of Risk Inform Patient Choice? Lessons From a Study of Prenatal Genetic Counseling,” Medical Anthropology 25, no. 3 (2006): 193–219. 16895827 10.1080/01459740600829720

[22] L. M. Schwartz , S. Woloshin , W. C. Black , and H. G. Welch , “The Role of Numeracy in Understanding the Benefit of Screening Mammography,” Annals of Internal Medicine 127, no. 11 (1997): 966–972. 9412301 10.7326/0003-4819-127-11-199712010-00003

[23] J. E. Corrarino , “Health Literacy and Women's Health: Challenges and Opportunities,” Journal of Midwifery & Women's Health 58, no. 3 (2013): 257–264. 10.1111/jmwh.12018 23631442

[24] F. Nawabi , F. Krebs , V. Vennedey , A. Shukri , L. Lorenz , and S. Stock , “Health Literacy in Pregnant Women: A Systematic Review,” International Journal of Environmental Research and Public Health 18, no. 7 (2021): 3847. 33917631 10.3390/ijerph18073847 PMC8038834

[25] Index of Multiple Deprivation 2019 A Liverpool Analysis Appendix B – Liverpool Ward Analysis (2019).

[26] NHS England , “Deprivation,” NHS England; [updated 2021], https://www.england.nhs.uk/about/equality/equality‐hub/national‐healthcare‐inequalities‐improvement‐programme/what‐are‐healthcare‐inequalities/deprivation/.

[27] NIHR , “Guidance on Co‐Producing a Research Project,” (2021), https://www.learningforinvolvement.org.uk/content/resource/nihr‐guidance‐on‐co‐producing‐a‐research‐project/.

[28] UK Research and Innovation , “Co‐production in research”.

[29] “Co‐Production: What It Is and How to Do It,” Social care institute for excellence. (2022).

[30] NHS England , “Co‐Production”.

[31] S. Redman , T. Greenhalgh , L. Adedokun , S. Staniszewska , and S. Denegri , “Co‐Production of Knowledge: The Future,” BMJ 372 (2021): n434. 33593753 10.1136/bmj.n434 PMC7884902

[32] A. Demetri , A. Davies , D. Bakhbakhi , et al., “Vaginal Birth Core Information Set: Study Protocol for a Delphi Study to Achieve a Consensus on a ‘Core Information Set’ for Vaginal Birth,” BMJ Open 13 (2023): e070215. 10.1136/bmjopen-2022-070215 PMC10407363 37550021

[33] G. Eysenbach , “Improving the Quality of Web Surveys: The Checklist for Reporting Results of Internet E‐Surveys (CHERRIES),” Journal of Medical Internet Research 6, no. 3 (2004): e34. 15471760 10.2196/jmir.6.3.e34 PMC1550605

[34] I. M. Lipkus , G. Samsa , and B. K. Rimer , “General Performance on a Numeracy Scale Among Highly Educated Samples,” Medical Decision Making 21, no. 1 (2001): 37–44. 11206945 10.1177/0272989X0102100105

[35] E. T. Cokely , M. Galesic , E. Schulz , S. Ghazal , and R. Garcia‐Retamero , “Measuring Risk Literacy: The Berlin Numeracy Test,” Judgment and Decision making 7, no. 1 (2012): 25–47.

[36] “Single‐Item Literacy Screener (SILS) Questionnaire,” (2022), https://www.codetechnology.com/blog/single‐item‐literacy‐screener‐sils‐questionnaire/.

[37] S. Dryhurst , J. Kerr , and A. L. J. Freeman , Method to Test the Effects of Different Formats of Communication of Indirect Uncertainty Around the COVID‐19 R Number on a Public Audience Octopus 2023.

[38] P. A. Harris , R. Taylor , B. L. Minor , et al., “The REDCap Consortium: Building an International Community of Software Platform Partners,” Journal of Biomedical Informatics 95 (2019): 103208. 31078660 10.1016/j.jbi.2019.103208 PMC7254481

[39] P. A. Harris , R. Taylor , R. Thielke , J. Payne , N. Gonzalez , and J. G. Conde , “Research Electronic Data Capture (REDCap)–A Metadata‐Driven Methodology and Workflow Process for Providing Translational Research Informatics Support,” Journal of Biomedical Informatics 42, no. 2 (2009): 377–381. 18929686 10.1016/j.jbi.2008.08.010 PMC2700030

[40] R Core Team , R: A Language and Environment for Statistical Computing (R Foundation for Statistical Computing, 2024).

[41] S. Y. D. Lee , B. D. Stucky , and J. Y. Lee , “Short Assessment of Health Literacy–Spanish and English: A Comparable Test of Health Literacy for Spanish and English Speakers,” Health Services Research 45, no. 4 (2010): 1105–1120. 20500222 10.1111/j.1475-6773.2010.01119.x PMC2910571

[42] B. D. Weiss , M. Z. Mays , and W. Martz , “Quick Assessment of Literacy in Primary Care: The Newest Vital Sign,” Annals of Family Medicine 3, no. 6 (2005): 514–522. 16338915 10.1370/afm.405 PMC1466931

[43] M. Patrick , R. D. Venkatesh , and D. R. Stukus , “Social Media and Its Impact on Health Care,” Annals of Allergy, Asthma & Immunology 128 (2022): 139–145. 10.1016/j.anai.2021.09.014 34555532

[44] R. Garcia‐Retamero and E. T. Cokely , “Designing Visual Aids That Promote Risk Literacy: A Systematic Review of Health Research and Evidence‐Based Design Heuristics,” Human Factors 59, no. 4 (2017): 582–627. 28192674 10.1177/0018720817690634

[45] J. P. Oudhoff and D. R. M. Timmermans , “The Effect of Different Graphical and Numerical Likelihood Formats on Perception of Likelihood and Choice,” Medical Decision Making 35, no. 4 (2015): 487–500. 25769496 10.1177/0272989X15576487

[46] “Planned Caesarean Birth (Consent Advice No. 14),” RCOG, https://www.rcog.org.uk/guidance/browse‐all‐guidance/consent‐advice/planned‐caesarean‐birth‐consent‐advice‐no‐14/.

