# Supplementary material for: How Should We Communicate Information Regarding Birth Choices to Women?: An Online Randomised Survey
Source: BJOG. 2025 Jul 28;132(13):2177–85. doi: 10.1111/1471-0528.18314 (PMC12592780; doi:10.1111/1471-0528.18314)
Supplement: Supplementary file 1 — Data S1: bjo18314_sup‐0001‐Appendix 1.docx. [file BJO-132-2177-s001.docx]

Appendix 1: Online Survey


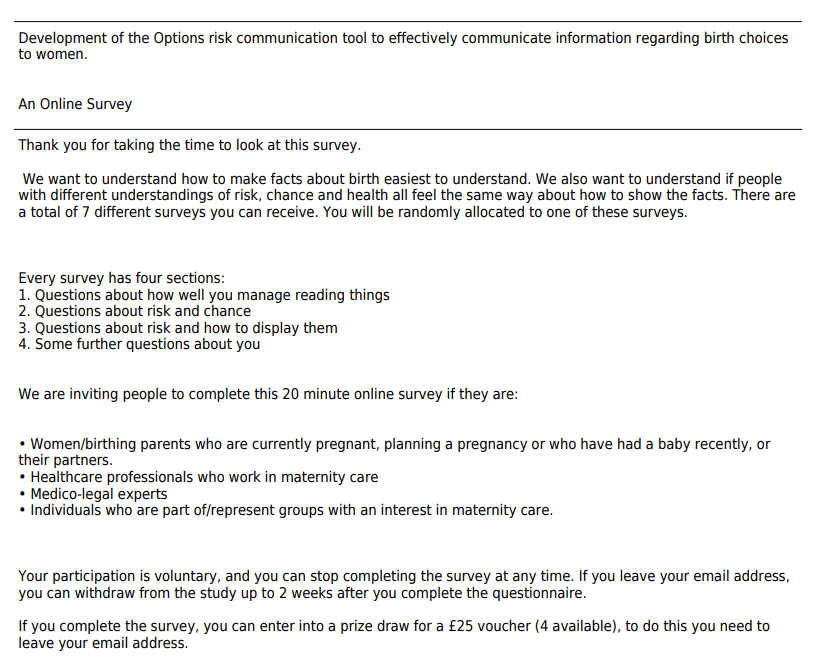


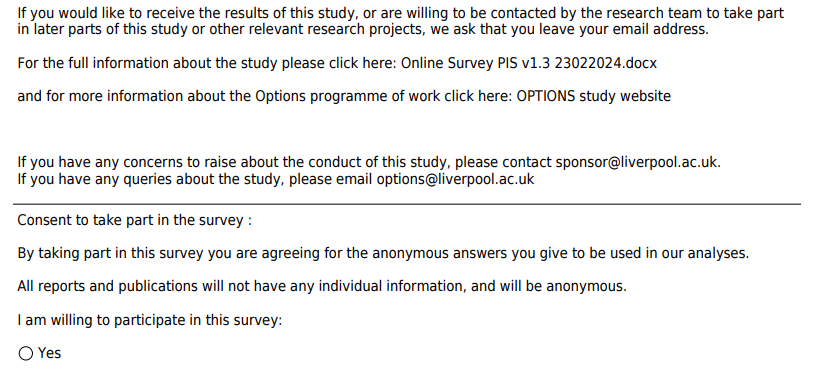


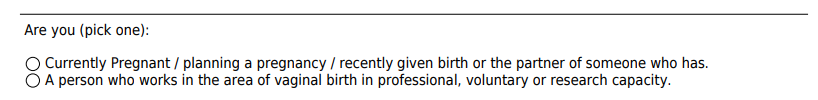

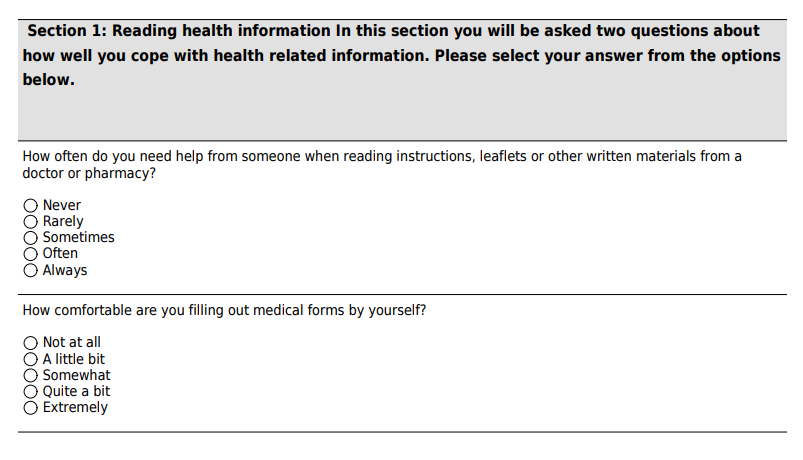

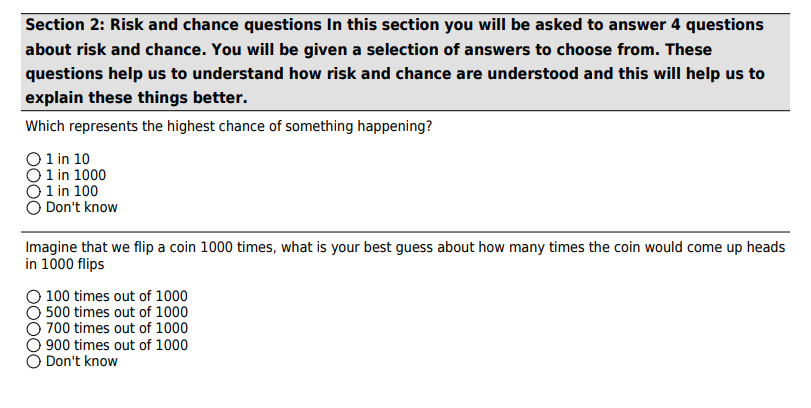

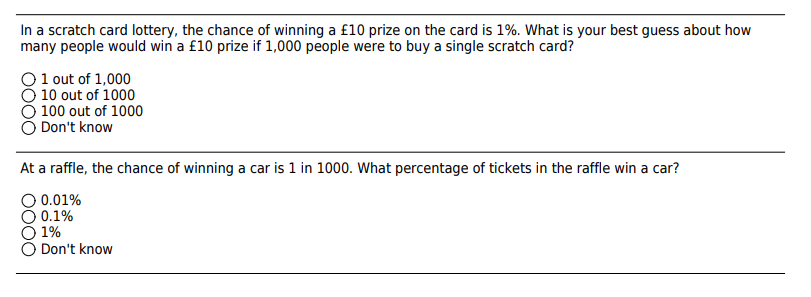

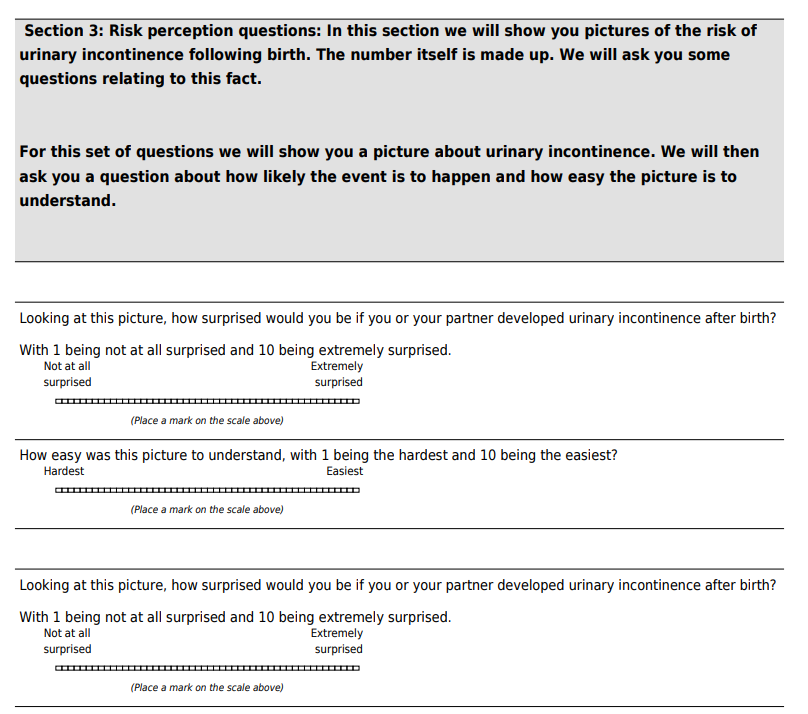

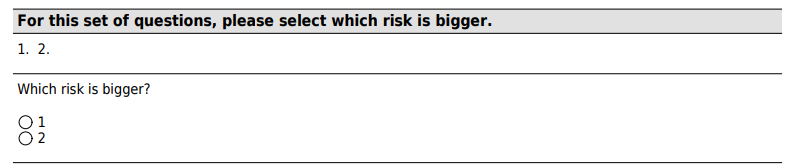

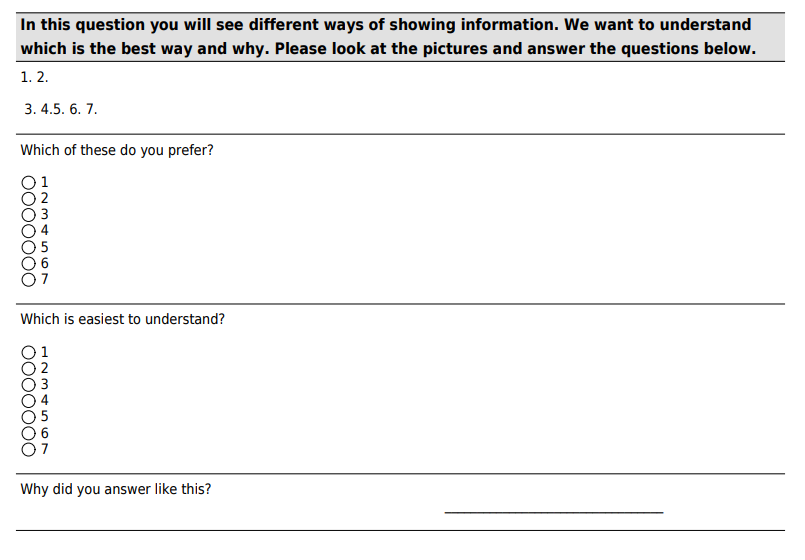

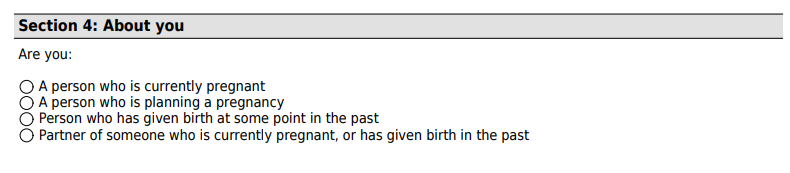

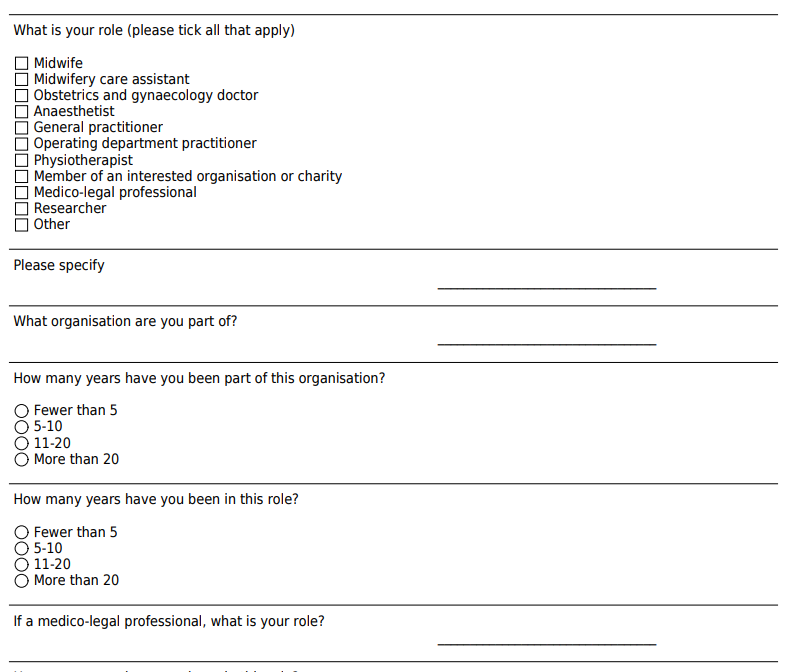

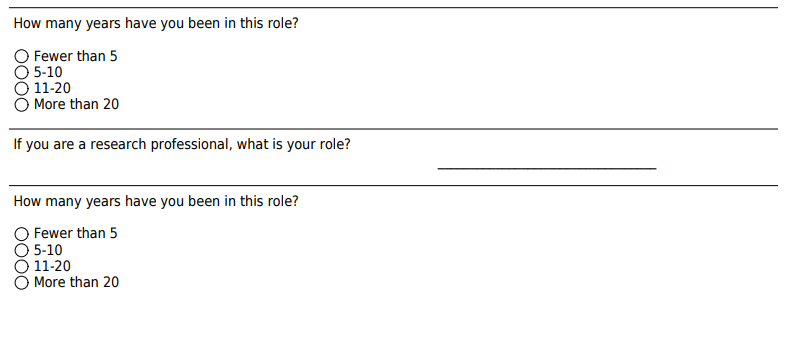

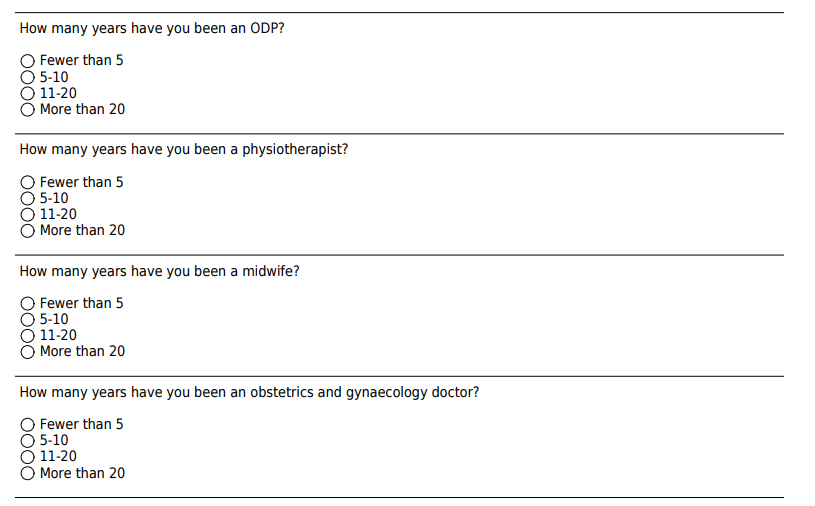

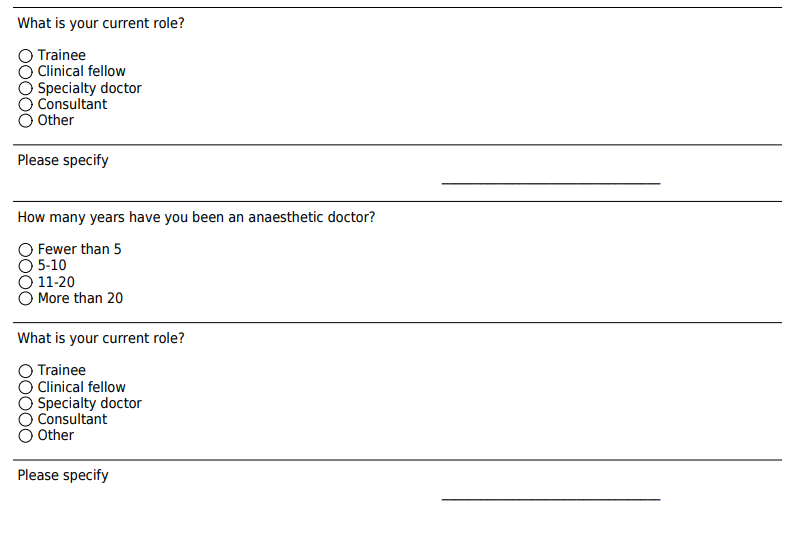

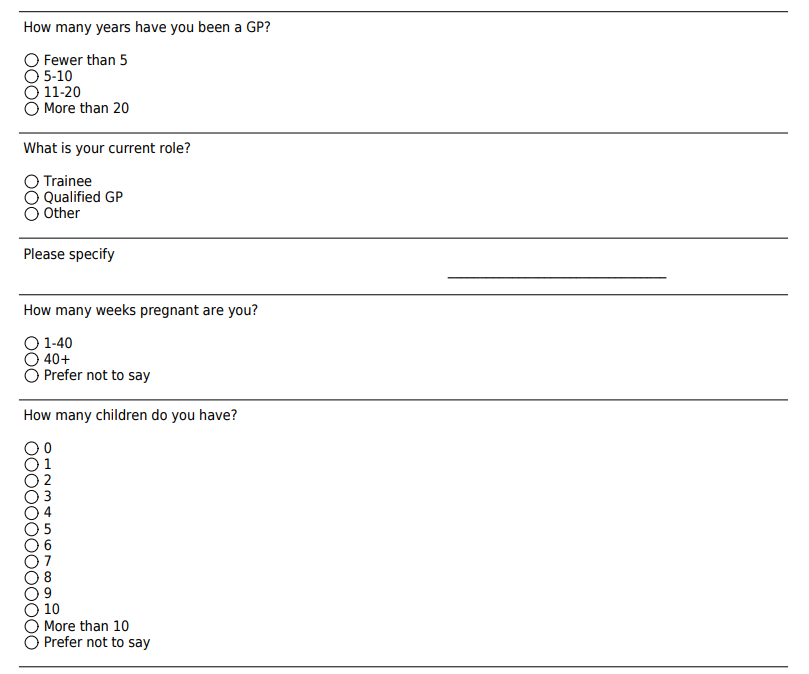

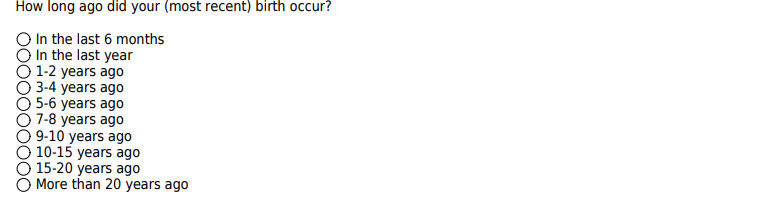

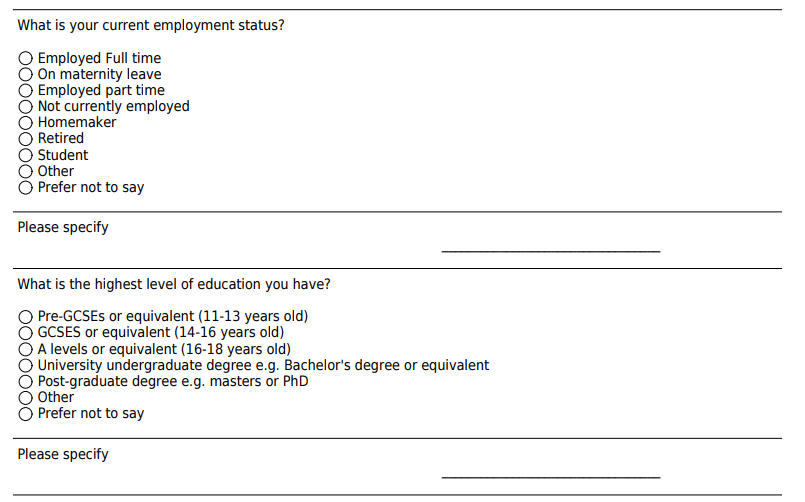

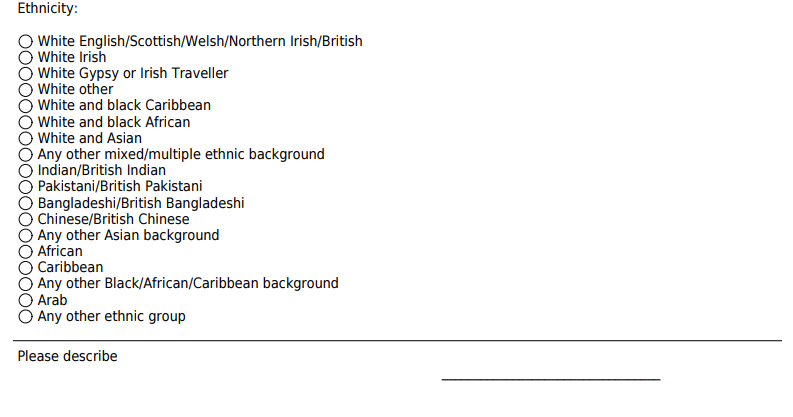

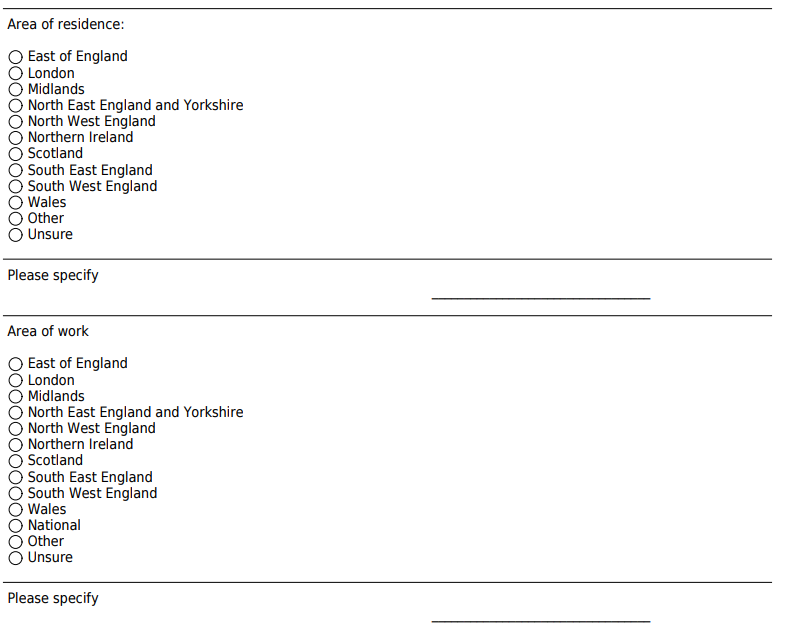

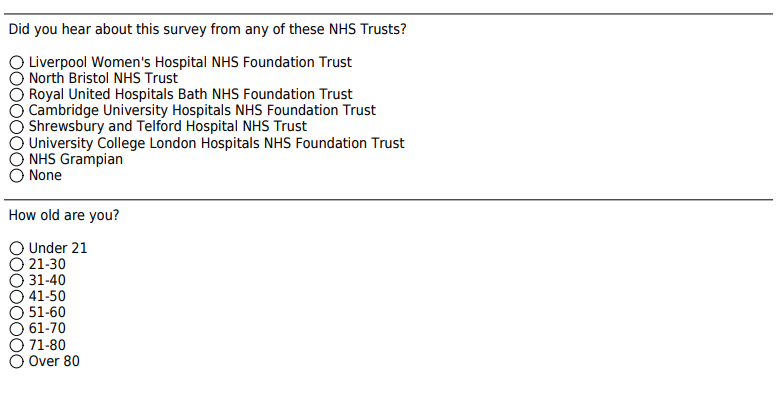

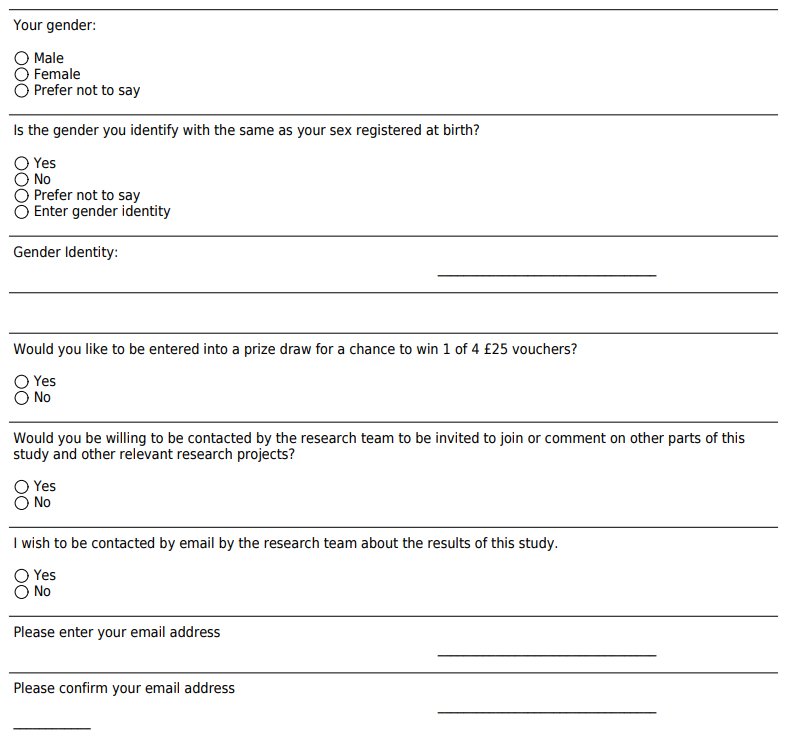


Appendix 2: Poster for the online survey


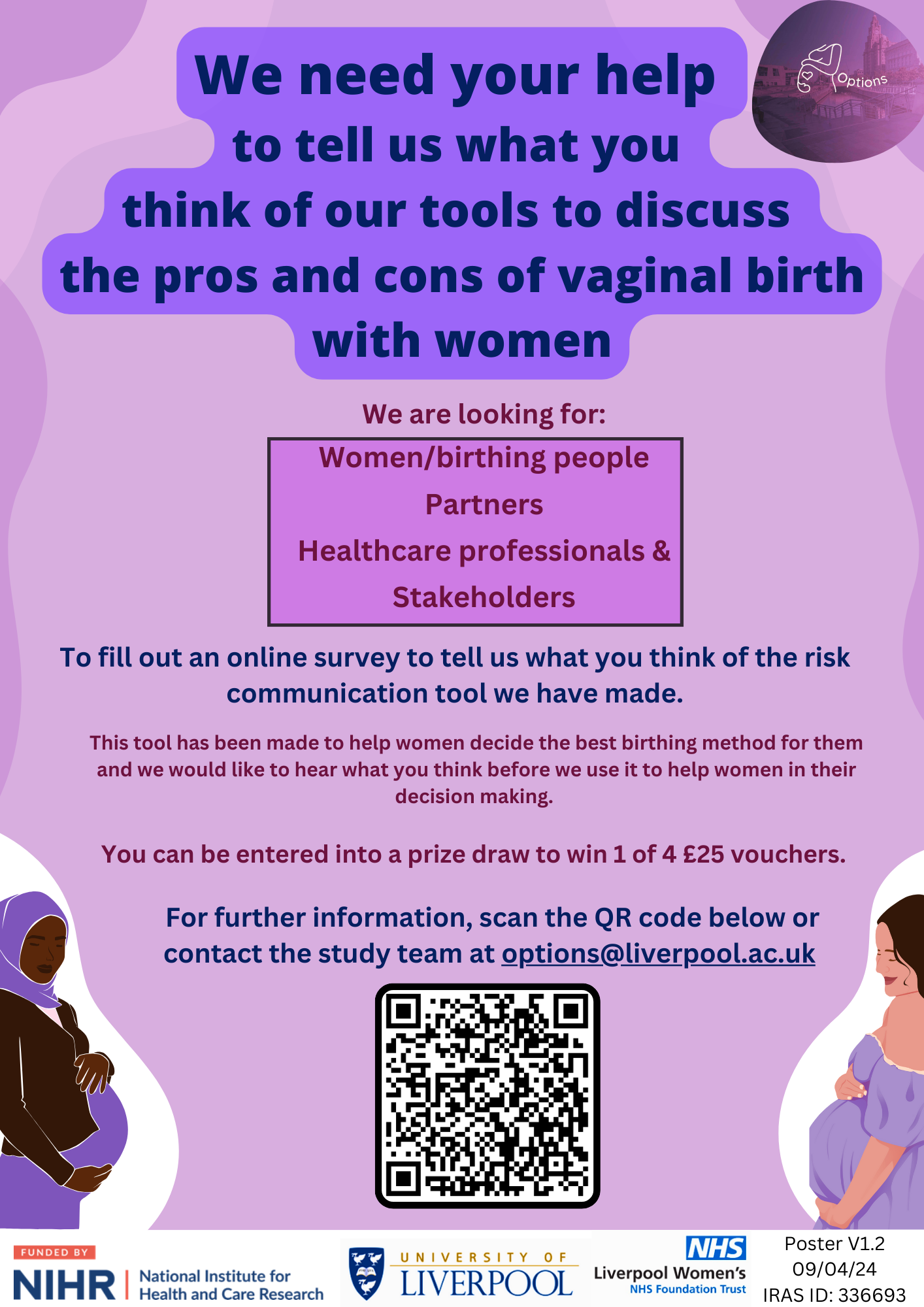


Appendix 3: Social media text:

***Facebook advert for social media (for pregnant women/birthing people /partners)***

Are you pregnant, planning a pregnancy or have you had a baby recently, or a partner of someone who has? If yes, we would like to hear from you! We are looking for participants to participate in an online survey. We have created some tools to help discuss the risks and benefits of vaginal birth and want to understand what you think of them and which are the best ones. The survey is online and will take approximately 30 minutes to complete. You can enter into a prize draw to win 1 of 4 £25 vouchers. If you would like to take part or for more information see [link]or email: [options@liverpool.ac.uk](mailto:options@liverpool.ac.uk)

Thank you!

***Twitter advert (for pregnant women):***

Are you pregnant, planning a pregnancy, recently had a baby or a partner? We need your help to understand the best ways to communicate information about vaginal birth. You can a prize draw 1 of four £25 vouchers. For more info: [insert link here]

***Facebook advert for social media and hospital groups (for healthcare professionals/stakeholders)***

[Are you a healthcare professional who works with pregnant women antenatally or postnatally? Or someone who supports women in decision making about birth or represents an organisation that does?]  If yes, we would like to hear from you! We are looking for participants for an online survey to tell us you think of our risk communication tools regarding vaginal birth. The survey will be carried out online and take approximately [x]minutes to complete. You can enter a prize draw to win 1 of 4 £25 vouchers. If you would like to take part or for more information, please see [survey link]or email: options@liverpool.ac.uk Thank you!

***Twitter advert (for healthcare professionals):***

Do you support women in making choices about vaginal birth? We need you to tell us what you think about our risk communication tool to discuss risks and benefits of vaginal birth to women. You can enter a £25 prize draw. For more info see [study link]

Appendix 4: Recruitment email

***Email recruitment text (Health Care Professionals/Organisation):***

Dear xxxx,

I am writing to you regarding the development of a tool to communicate information, including benefits and risks of vaginal birth.

We are seeking the input from [Health care professionals/stakeholders including medicolegal experts/organisations who advocate for women’s rights/birthing information].

We are therefore inviting you/members of your organisation, to participate in a short online survey. The survey is online and will take approximately [x] minutes to complete.

If you complete the surveys, you can if you choose be entered into a prize draw for 1 of 4 £25 vouchers.

Please see [survey link] for more information or contact us on email: [options@liverpool.ac.uk](mailto:options@liverpool.ac.uk)

We would be grateful if you could share this link within your networks.

Appendix 5:

Impressions from social media recruitment:

| Social media site | Number of posts | Number of people reached | Number of clicks to the survey | Likes/Reactions | Comments | Shares |
| --- | --- | --- | --- | --- | --- | --- |
| Facebook | 6 | 17,209 | 581 | 33 | 33 | 13 |
| X | 4 | 4,600 | Unknown | 34 | 0 | 28 |
| Instagram | 1 | 2312 | 72 | 7 | 2 | 4 |
