# Supplementary material for: How Should We Communicate Information Regarding Birth Choices to Women?: An Online Randomised Survey
Source: BJOG. 2025 Jul 28;132(13):2177–85. doi: 10.1111/1471-0528.18314 (PMC12592780; doi:10.1111/1471-0528.18314)
Supplement: Supplementary file 2 — Data S2: bjo18314_sup‐0002‐Supplementary‐Figures‐Tables.docx. [file BJO-132-2177-s002.docx]

**Supplementary file of Figures and Tables:**


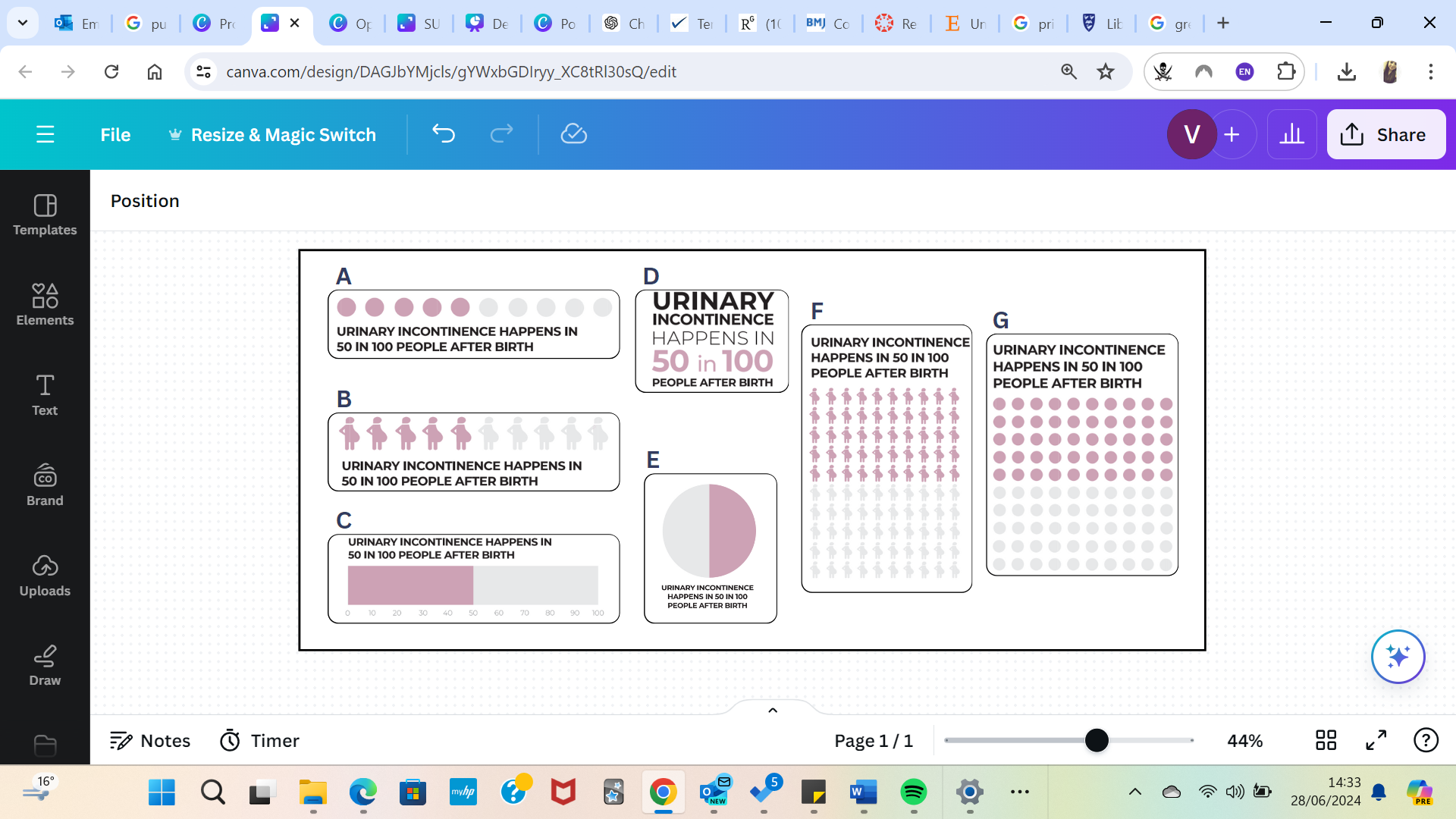


Supplementary figure 1: The seven types of graphics tested within the survey. A) 10 circles icon array, B) 10 people icon array, C) Bar chart, D) Words, E) Pie chart, F) 100 people icon array, G) 100 circles icon array.

The risk of 50 in 100 women having urinary incontinence after birth is displayed in seven different ways. These methods of presenting risk were chosen alongside PPI input to take forward into the survey. Graphic A uses 10 circles to represent 100 people with half shaded. Graphic B uses 10 people to represent the same risk. Graphic C uses a bar chart format where half of the bar is shaded in. Graphic D shows the risk with exclusively words and no adjunctive graphic alongside. Graphic E shows a pie chart to represent the total of 100 people with half shaded. Graphic F shows 100 people with 50 shaded in. Graphic G is similar to graphic F but rather than people, uses circles.

The risk of urinary incontinence after birth was chosen following PPI involvement. They highlighted the importance of understanding risks of birth not only during, but the longer-term consequences. They highlighted how the future implications of a birth can influence their decision-making regarding mode of birth. They felt that urinary incontinence fit the concept of ‘surrounding’ birth.


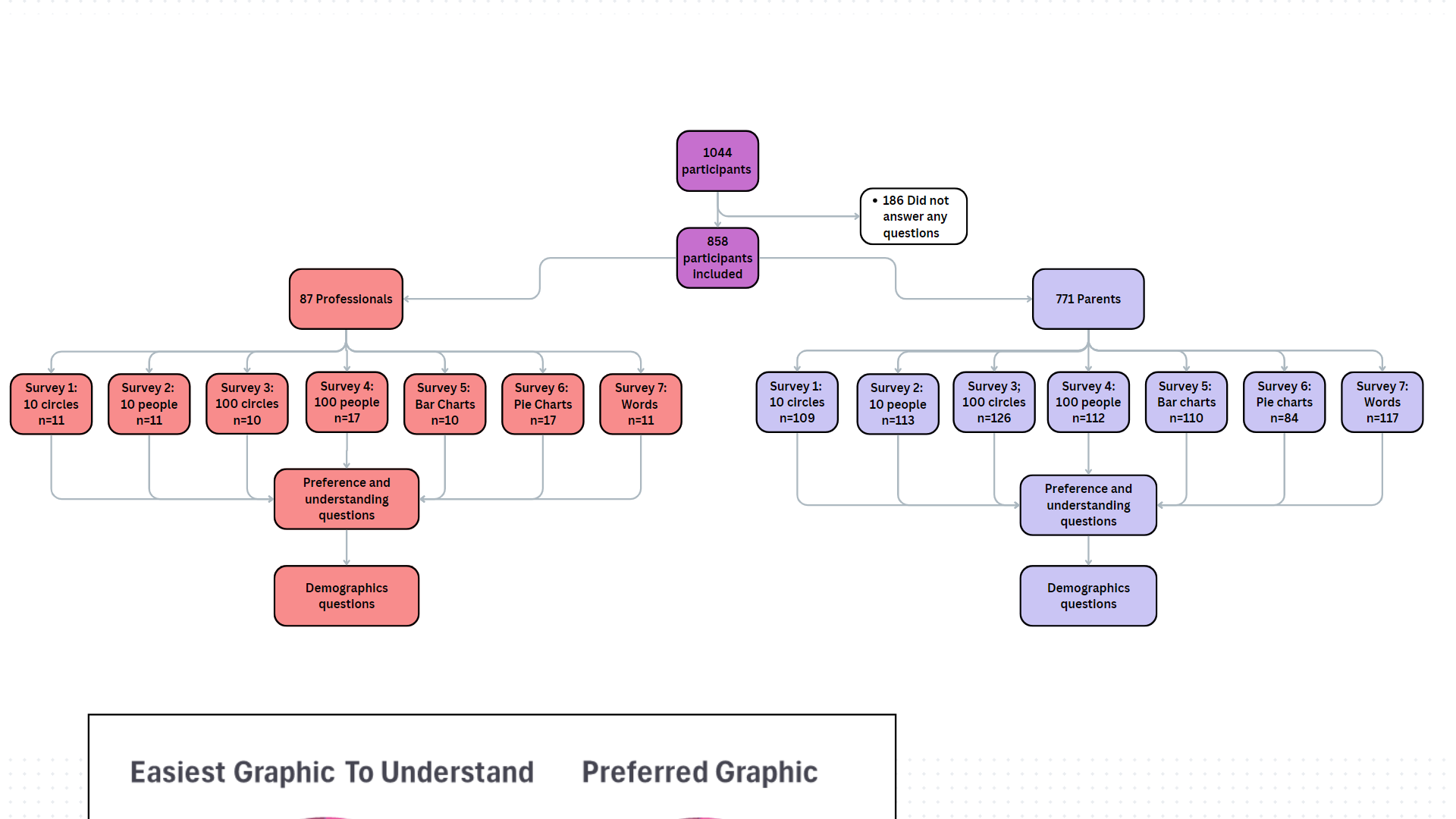
 Supplementary figure 2: Flow diagram for the random allocation of the surveys, stratified by parents and professionals demonstrating the number of included participants within each survey arm and reasons for exclusion.

Supplementary Table 1: Demographics and characteristics of participants by parent and professional status.

|  | **Professionals**  **(n=87)** | **Parents**  **(n=771)** | **Total**  **(n=858)** |
| --- | --- | --- | --- |
| **Age (%)** | | | |
| Under 21 | 0 | 2 (0%) | 2 (0%) |
| 21-30 | 15 (17%) | 174 (23%) | 189 (22%) |
| 31-40 | 41 (47.1%) | 555 (72.0%) | 596 (69.5%) |
| 41-50 | 17 (19.5%) | 38 (4.9%) | 55 (6.4%) |
| 51-60 | 12 (13.8%) | 1 (0.1%) | 13 (1.5%) |
| 61-70 | 2 (2.3%) | 0 | 2 (0.2%) |
| Unknown | 0 | 1 (0.1%) | 1 (0.1%) |
| **Sex (%)** | | | |
| Male | 3 (3.4%) | 9 (1.1%) | 12 (1.4%) |
| Female | 83 (95.4%) | 747 (96.9%) | 830 (96.7%) |
| Prefer not to say | 1 (1.1%) | 3 (1.3%) | 4 (0.5%) |
| Unknown | 0 | 5 (0.6%) | 5 (0.6%) |
| **Ethnicity (%)** | | | |
| White English/Scottish/Welsh/Northern Irish/British | 66 (75.8%) | 638 (82.7%) | 704 (82.1%) |
| White Irish | 4 (4.6%) | 36 (4.7%) | 40 (4.7%) |
| White Gypsy or Irish Traveller | 1 (1.1%) | 0 | 1 (0.12%) |
| White other | 9 (10.3%) | 50 (6.5) | 59 (6.9%) |
| White and Black Caribbean | 0 | 4 (0.5%) | 4 (0.5%) |
| White and Black African | 0 | 4 (0.5%) | 4 (0.5%) |
| White and Asian | 1 (1.1%) | 8 (1.0%) | 9 (1.0%) |
| Any other mixed/multiple ethnic background | 2 (2.3%) | 3 (0.4%) | 5 (0.6.%) |
| Indian/British Indian | 0 | 8 (1.04%) | 8 (0.93%) |
| Pakistani/British Pakistani | 0 | 1 (0.13%) | 1 (0.12%) |
| Chinese/British Chinese | 3 (3.4%) | 4 (0.52%) | 7 (0.8%) |
| Any other Asian background | 0 | 2 (0.3%) | 2 (0.2%) |
| African | 1 (1.1%) | 2 (0.3%) | 3 (0.3%) |
| Caribbean | 0 | 1 (0.1%) | 1 (0.1%) |
| Any other Black/African/Caribbean background | 0 | 2 (0.1%) | 2 (0.2%) |
| Any other ethnic group | 0 | 2 (0.3%) | 2 (0.23%) |
| Unknown | 0 | 6 (0.8%) | 6 (0.7%) |
| **Highest Level of Education (%)** | | | |
| Pre-GCSEs or equivalent | 1 (1.1%) | 3 (0.4%) | 4 (0.47%) |
| GCSES or equivalent | 0 | 20 (2.6%) | 20 (2.3%) |
| A level or equivalent | 4 (4.6%) | 74 (9.6%) | 78 (9.1%) |
| University undergraduate degree | 49 (56.3%) | 342 (44.4%) | 391 (45.6%) |
| Post-graduate degree | 31 (35.6%) | 316 (41.0%) | 347 (40.4%) |
| Other | 1 (1.1%) | 12 (1.56%) | 13 (1.5%) |
| Prefer not to say | 1 (1.1%) | 3 (0.4%) | 4 (0.5%) |
| Unknown | 0 | 1 (0.1%) | 1 (0.1%) |
| **Employment status (%)** | | | |
| Employed full-time | 49 (56.0%) | 238 (31%) | 287 (33.4%) |
| On maternity leave | 7 (8.0%) | 299 (38.8%) | 306 (35.7%) |
| Employed part-time | 25 (28.7%) | 143 (18.5%) | 168 (19.6%) |
| Not currently employed | 1 (1.1%) | 12 (1.6%) | 13 (1.5%) |
| Homemaker | 1 (1.1%) | 57 (7.4%) | 58 (6.8%) |
| Retired | 0 | 1 (0.1%) | 1 (0.1%) |
| Student | 1 (1.1%) | 8 (1.0%) | 9 (1.0%) |
| Other | 2 (2.3%) | 13 (1.7%) | 15 (1.7%) |
| Prefer not to say | 1 (1.1%) | 0 | 1 (0.1%) |
| **Area of residence (%)** | | | |
| East of England | 13 (15%) | 75 (9.7%) | 88 (10.3%) |
| London | 2 (2.3%) | 66 (8.6%) | 68 (7.9%) |
| Midlands | 18 (20.7%) | 104 (13.5%) | 122 (14.2%) |
| North East England | 7 (8.0%) | 94 (12.2%) | 101 (11.8%) |
| Yorkshire | 19 (21.8%) | 92 (11.9%) | 111 (12.9%) |
| North West England | 6 (6.9%) | 33 (4.3%) | 39 (4.5%) |
| Northern Ireland | 7 (8.0%) | 79 (10.2%) | 86 (10.0%) |
| Scotland | 7 (8.0%) | 92 (11.9%) | 99 (11.5%) |
| South East England | 6 (6.9%) | 89 (11.5%) | 95 (11.1%) |
| South West England | 2 (2.3%) | 39 (5.1%) | 41 (4.8%) |
| Wales | 0 | 5 (0.6%) | 5 (0.6%) |
| Other | 0 | 2 (0.3%) | 2 (0.2%) |
| Unknown | 0 | 1 (0.1%) | 1 (0.1%) |
| **Pregnant, planning a pregnancy, previously given birth, a partner or unknown (%)** | | | |
| Currently pregnant |  | 200 (25.9%) |  |
| Planning a pregnancy |  | 52 (6.7%) |  |
| Previously given birth |  | 502 (65.1%) |  |
| Given birth within the last 6 months |  | 225 (44.8%) |  |
| Within the last year |  | 116 (23.1%) |  |
| 1-2 years ago |  | 127 (25.3%) |  |
| 3-4 years ago |  | 24 (4.8%) |  |
| 5-6 years ago |  | 6 (1.2%%) |  |
| ≥ 7 years ago |  | 4 (0.8%) |  |
| Partner |  | 14 (1.8%) |  |
| Unknown |  | 3 (0.4%) |  |
| **Number of children (%)** | | | |
| 0 |  | 93 (12.1%) |  |
| 1 |  | 361 (46.8%) |  |
| 2 |  | 260 (33.7%) |  |
| 3 |  | 32 (4.2%) |  |
| 4 |  | 14 (1.8%) |  |
| ≥5 |  | 11 (1.4%) |  |
| **Role of professionals (%)** | | | |
| Midwife | 46 (52.9%) |  |  |
| Midwifery care assistant | 4 (4.6%) |  |  |
| Obstetrics and gynaecology doctor | 18 (20.7%) |  |  |
| Anaesthetist | 0 |  |  |
| General Practitioner | 3 (3.4%) |  |  |
| Operating Department Practitioner | 0 |  |  |
| Physiotherapist | 0 |  |  |
| Member of an interested organisation or charity | 5 (5.7%) |  |  |
| Medico-legal professional | 0 |  |  |
| Researcher | 3 (3.4%) |  |  |
| Other | 8 (9.2%) |  |  |

Supplementary table 2: Characteristics of participants within each survey group by parent and professional.

|  | **Survey 1** | | **Survey 2** | | **Survey 3** | | **Survey 4** | | **Survey 5** | | **Survey 6** | | | **Survey 7** | | |
| --- | --- | --- | --- | --- | --- | --- | --- | --- | --- | --- | --- | --- | --- | --- | --- | --- |
|  | **Parents**  **N=109** | **Professionals**  **N=11** | **Parents**  **N=113** | **Professionals**  **N=11** | **Parents**  **N=126** | **Professionals N=10** | **Parents**  **N=112** | **Professionals**  **N=17** | **Parents**  **N=110** | **Professionals**  **N=10** | **Parents**  **N=84** | **Professionals**  **N=17** | **Parents**  **N=117** | | **Professionals**  **N=11** |  |
| **Age (%)** | | | | | | | | | | | | | | | |  |
| Under 21 | 0 | 0 | 0 | 0 | 0 | 0 | 0 | 0 | 0 | 0 | 0 | 0 | 2 (2%) | | 0 |  |
| 21-30 | 23 (21%) | 1 (9%) | 24 (21%) | 2 (18%) | 37 (29%) | 1 (10%) | 22 (20%) | 3 (18%) | 26 (24%) | 1 (10%) | 22 (26%) | 4 (23%) | 20 (17%) | | 3 (27%) |  |
| 31-40 | 82 (75%) | 9 (82%) | 83 (73%) | 5 (45%) | 81 (64%) | 4 (40%) | 88 (79%) | 9 (53%) | 73 (66%) | 5 (50%) | 57 (68%) | 5 (29%) | 91 (78%) | | 4 (36%) |  |
| 41-50 | 4 (4%) | 1 (9%) | 6 (5%) | 1 (9%) | 8 (6%) | 1 (10%) | 2 (2%) | 3 (18%) | 11 (10%) | 3 (30%) | 3 (4%) | 5 (29%) | 4 (3%) | | 3 (27%) |  |
| 51-60 | 0 | 0 | 0 | 2 (18%) | 0 | 3 (30%) | 0 | 2 (12%) | 0 | 1 (10%) | 1 (1%) | 3 (18%) | 0 | | 1 (9%) |  |
| 61-70 | 0 | 0 | 0 | 1 (9%) | 0 | 1 (10%) | 0 | 0 | 0 | 0 | 0 | 0 | 0 | | 0 |  |
| Unknown | 0 | 0 | 0 | 0 | 0 | 0 | 0 | 0 | 0 | 0 | 1 (1%) | 0 | 0 | | 0 |  |
| **Sex** | | | | | | | | | | | | | | | |  |
| Male | 1 (1%) | 0 | 0 | 0 | 4 (3%) | 1 (10%) | 3 (3%) | 1 (6%) | 1 (1%) | 0 | 1 (1%) | 1 (6%) | 2 (2%) | | 0 |  |
| Female | 108 (99%) | 11 (100%) | 111 (98%) | 11 (100%) | 121 (96%) | 9  (90%) | 106 (95%) | 16 (94%) | 105 (95%) | 10 (100%) | 83  (99%) | 15  (88%) | 113 (97%) | | 11 (100%) |  |
| **Ethnicity** | | | | | | | | | | | | | | | |  |
| White English/Scottish/Welsh/Northern Irish/British | 90 (83%) | 8 (73%) | 93 (82%) | 8 (73%) | 100 (79%) | 10 (100%) | 98 (88%) | 12 (71%) | 91 (83%) | 6 (60%) | 60 (71%) | 15 (88%) | 106 (91%) | | 7 (64%) |  |
| White Irish | 5 (5%) | 1 (9%) | 9 (8%) | 1 (9%) | 5 (4%) | 0 | 3 (3%) | 1 (6%) | 7 (6%) | 0 | 3 (4%) | 0 | 4 (3%) | | 1 (9%) |  |
| White Gypsy | 0 | 0 | 0 | 0 | 0 | 0 | 0 | 0 | 0 | 1 (10%) |  | 0 | 0 | | 0 |  |
| White other | 4 (4%) | 0 | 5 (4%) | 0 | 10 (8%) | 0 | 6 (5%) | 3 (18%) | 9 (8%) | 3 (30%) | 10 (12%) | 1 (6%) | 6 (5%) | | 2 (18%) |  |
| White and Black Caribbean | 1 (1%) | 0 | 0 | 0 | 0 | 0 | 2 (2%) | 0 | 0 | 0 | 1 (1%) | 0 | 0 | | 0 |  |
| White and Black African | 1 (1%) | 0 | 3 (3%) | 0 | 0 | 0 | 0 | 0 | 0 | 0 | 0 | 0 | 0 | | 0 |  |
| White and Asian | 1 (1%) | 0 | 0 | 0 | 1 (1%) | 0 | 1 (1%) | 0 | 1 (1%) | 0 | 3 (4%) | 0 | 1 (1%) | | 1 (9%) |  |
| Any other mixed/multiple ethnic background | 0 | 2 (18%) | 0 | 0 | 1 (1%) | 0 | 1 (1%) | 0 | 0 | 0 | 1 (1%) | 0 | 0 | | 0 |  |
| Indian/British Indian | 1 (1%) | 0 | 2 (2%) | 0 | 3 (2%) | 0 | 0 | 0 | 0 | 0 | 2 (2%) | 0 | 0 | | 0 |  |
| Pakistani/British Pakistani | 0 | 0 | 1 (1%) | 0 | 0 | 0 | 0 | 0 | 0 | 0 | 0 | 0 | 0 | | 0 |  |
| Chinese/British Chinese | 0 | 0 | 0 | 2 (18%) | 2 (2%) | 0 | 0 | 0 | 1 (1%) | 0 | 1 (1%) | 1 (6%) | 0 | | 0 |  |
| Any other Asian Background | 0 | 0 | 0 | 0 | 0 | 0 | 0 | 0 | 1 (1%) | 0 | 1 (1%) | 0 | 0 | | 0 |  |
| African | 1 (1%) | 0 | 0 | 0 | 0 | 0 | 0 | 1 (6%) | 0 | 0 | 1 (1%) | 0 | 0 | | 0 |  |
| Caribbean | 0 | 0 | 0 | 0 | 1 (1%) | 0 | 0 | 0 | 0 | 0 | 0 | 0 | 0 | | 0 |  |
| Any other Black/African/Caribbean background. | 1 (1%) | 0 | 0 | 0 | 0 | 0 | 0 | 0 | 0 | 0 | 1 (1%) | 0 | 0 | | 0 |  |
| Any other ethnic group | 0 | 0 | 0 | 0 | 1 | 0 | 0 | 0 | 1 | 0 | 0 | 0 | 0 | | 0 |  |
| **Highest level of education** | | | | | | | | | | | | | | | |  |
| Pre GCSEs or equivalent | 0 | 0 | 1 (1%) | 0 | 2 (2%) | 0 | 0 | 1 (6%) | 0 | 0 | 0 | 0 | 0 | | 0 |  |
| GCSEs or equivalent | 3 (3%) | 0 | 2 (2%) | 0 | 9 (7%) | 0 | 0 | 0 | 2 (2%) | 0 | 3 (4%) | 0 | 1 (1%) | | 0 |  |
| A level of equivalent | 12 (11%) | 1 (9%) | 10 (9%) | 2 (18%) | 8 (6%) | 1 (10%) | 11 (10%) | 0 | 12 (11%) | 0 | 6 (7%) | 0 | 15 (13%) | | 0 |  |
| University undergraduate degree | 44 (40%) | 8 (73%) | 42 (37%) | 6 (55%) | 53 (42%) | 4 (40%) | 60 (54%) | 9 (53%) | 54 (49%) | 7 (70%) | 41 (49%) | 7 (41%) | 48 (41%) | | 8 (73%) |  |
| Post-graduate degree | 48 (44%) | 2 (18%) | 57 (50%) | 3 (27%) | 54 (43%) | 5 (50%) | 39 (35%) | 7 (41%) | 40 (36%) | 2 (20%) | 31 (37%) | 10 (59%) | 47 (40%) | | 2 (18%) |  |
| Other | 1 (1%) | 0 | 1 (1%) | 0 | 0 | 0 | 1 (1%) | 0 | 2 (2%) | 1 (10%) | 2 (2%) | 0 | 5 (4%) | | 0 |  |
| Prefer not to say | 1 (1%) | 0 | 0 | 0 | 0 | 0 | 1 (1%) | 0 | 0 | 0 | 1 (1%) | 0 | 1 (1%) | | 0 |  |
| Unknown | 0 | 0 | 0 | 0 | 0 | 0 | 0 | 0 | 0 | 0 | 0 | 0 | 0 | | 1 (9%) |  |
| **Employment status** | | | | | | | | | | | | | | | |  |
| Employed full time | 38 (35%) | 7 (64%) | 30 (27%) | 7 (64%) | 37 (29%) | 6 (60%) | 35 (31%) | 10 (59%) | 29 (26%) | 3 (30%) | 31 (37%) | 10 (59%) | 38 (32%) | | 6 (55%) |  |
| On maternity leave | 43 (39%) | 0 | 40 (35%) | 0 | 61 (48%) | 1 (10%) | 47 (42%) | 1 (6%) | 36 (33%) | 3 (30%) | 33 (39%) | 1 (6%) | 39 (33%) | | 1 (9%) |  |
| Employed part time | 19 (17%) | 3 (27%) | 24 (21%) | 2 (18%) | 15 (12%) | 3 (30%) | 20 (17%) | 5 (29%) | 28 (25%) | 4 (40%) | 15 (18%) | 5 (29%) | 22 (19%) | | 4 (36%) |  |
| Not currently employed | 2 (2%) | 1 (9%) | 2 (2%) | 0 | 2 (2%) | 0 | 2 (2%) | 0 | 2 (2%) | 0 | 4 (5%) | 0 | 2 (2%) | | 0 |  |
| Homemaker | 5 (5%) | 0 | 13 (12%) | 0 | 8 (6%) | 0 | 7 (6%) | 1 (6%) | 8 (7%) | 0 | 0 | 0 | 12 (10%) | | 0 |  |
| Retired | 0 | 0 | 1 (1%) | 0 | 0 | 0 | 0 | 0 | 0 | 0 | 1 (1%) | 0 | 4 (3%) | | 0 |  |
| Student | 0 | 0 | 2 (2%) | 1 (9%) | 0 | 0 | 1 (1%) | 0 | 4 (4%) | 0 | 0 | 0 | 0 | | 0 |  |
| Other | 2 (2%) | 1 (9%) | 1 (1%) | 1 (9%) | 3 (2%) | 0 | 0 | 0 | 3 (3%) | 0 | 0 | 0 | 0 | | 0 |  |
| Prefer not to say | 0 |  | 0 | 0 | 0 | 0 | 0 | 0 | 0 | 0 | 0 | 1 (6%) | 0 | | 0 |  |
| **Area of residence** | | | | | | | | | | | | | | | |  |
| East of England | 10 (9%) | 1 (9%) | 14 (12%) | 2 (18%) | 12 (10%) | 4 (40%) | 11 (10%) | 2 (12%) | 6 (5%) | 0 | 11 (13%) | 3 (18%) | 11 (9%) | | 1 (9%) |  |
| London | 10 (9%) | 2 (18%) | 9 (8%) | 0 | 15 (12%) | 0 | 4 (4%) | 4 (24%) | 8 (7%) | 0 | 9 (11%) | 0 | 11 (9%) | | 0 |  |
| Midlands | 13 (12%) | 1 (9%) | 14 (12%) | 2 (18%) | 24 (19%) | 2 (20%) | 7 (6%) | 1 (6%) | 12 (11%) | 2 (20%) | 12 (14%) | 5 (29%) | 22 (19%) | | 2 (18%) |  |
| North East England | 13 (12%) | 1 (9%) | 16 (14%) | 0 | 10 (8%) | 0 | 19 (17%) | 3 (18%) | 15 (14%) | 2 (20%) | 8 (10%) | 1 (6%) | 13 (11%) | | 2 (18%) |  |
| Yorkshire | 16 (15%) | 3 (27%) | 7 (6%) | 3 (27%) | 11 (9%) | 2 (20%) | 19 (17%) | 1 (6%) | 12 (11%) | 3 (30%) | 11 (13%) | 3 (18%) | 16 (14%) | | 2 (18%) |  |
| North West England | 4 (4%) | 0 | 4 (4%) | 1 (9%) | 7 (6%) | 0 | 5 (4%) | 4 (24%) | 6 (5%) | 0 | 2 (2%) | 1 (6%) | 5 (4%) | | 3 (27%) |  |
| Northern Ireland | 5 (5%) | 1 (9%) | 12 (11%) | 1 (9%) | 11 (9%) | 0 | 13 (12%) | 0 | 20 (18%) | 0 | 8 (10%) | 1 (6%) | 9 (8%) | | 0 |  |
| Scotland | 15 (14%) | 1 (9%) | 19 (17%) | 1 (9%) | 12 (10%) | 2 (20%) | 14 (13%) | 1 (6%) | 14 (12%) | 1 (10%) | 12 (14%) | 2 (12%) | 6 (5%) | | 0 |  |
| South East England | 12 (11%) | 1 (9%) | 10 (9%) | 1 (9%) | 16 (13%) | 0 | 13 (12%) | 1 (6%) | 12 (11%) | 2 (20%) | 9 (11%) | 0 | 17 (15%) | | 1 (9%) |  |
| South West England | 8 (9%) | 0 | 8 (7%) | 0 | 4 (3%) | 0 | 5 (4%) | 0 | 5 (5%) | 0 | 2 (2%) | 1 (6%) | 7 (6%) | | 0 |  |
| Wales | 1 (1%) | 0 | 0 | 0 | 2 (2%) | 0 | 2 (2%) | 0 | 0 | 0 | 0 | 0 | 0 | | 0 |  |
| Other | 1 (1%) | 0 | 0 | 0 | 2 (2%) | 0 | 0 | 0 | 0 | 0 | 0 | 0 | 0 | | 0 |  |
| **Pregnant, planning a pregnancy, previously given birth, or a partner** | | | | | | | | | | | | | | | |  |
| Currently pregnant | 28 (26%) |  | 31 (27%) |  | 26 (21%) |  | 33 (29%) |  | 25 (23%) |  | 28 (33%) |  | 29 (25%) | |  |  |
| Planning a pregnancy | 6 (6%) |  | 8 (7%) |  | 4 (3%) |  | 6 (5%) |  | 12 (11%) |  | 11 (13%) |  | 5 (4%) | |  |  |
| Previously given birth | 73 (67%) |  | 71 (63%) |  | 91 (72%) |  | 72 (64%) |  | 71 (65%) |  | 44 (52%) |  | 80 (68%) | |  |  |
| Partner | 2 (2%) |  | 3 (3%) |  | 4 (3%) |  | 1 (1%) |  | 2 (2%) |  | 0 |  | 2 (2%) | |  |  |
| Unknown | 0 |  | 0 |  | 0 |  | 0 |  | 0 |  | 1 (1%) |  | 1 (1%) | |  |  |
| **Number of children** | | | | | | | | | | | | | | | |  |
| 0 | 12 (11%) |  | 13 (12%) |  | 12 (10%) |  | 20 (18%) |  | 13 (12%) |  | 13 (15%) |  | 10 (9%) | |  |  |
| 1 | 43 (40%) |  | 58 (51%) |  | 65 (52%) |  | 53 (47%) |  | 53 (48\5) |  | 35 (42%) |  | 54 (46%) | |  |  |
| 2 | 46 (42%) |  | 33 (29%) |  | 38 (30%) |  | 32 (29%) |  | 34 (31%) |  | 29 (35%) |  | 48 (41%) | |  |  |
| 3 | 3 (3%) |  | 6 (5%) |  | 7 (6%) |  | 5 (4%) |  | 5 (5%) |  | 3 (4%) |  | 3 (3%) | |  |  |
| 4 | 3 (3%) |  | 3 (3%) |  | 3 (2%) |  | 1 (1%) |  | 2 (2%) |  | 3 (4%) |  | 2 (2%) | |  |  |
| ≥5 | 2 (2%) |  | 0 |  | 1 (1%) |  | 1 (1%) |  | 3 (3%) |  | 1 (1%) |  | 0 | |  |  |
| **Role of professionals** | | | | | | | | | | | | | | | |  |
| Midwife |  | 5 (45%) |  | 4 (36%) |  | 6 (60%) |  | 9 (53%) |  | 7 (70%) |  | 9 (53%) |  | | 6 (55%) |  |
| Midwifery Care assistant |  | 1 (9%) |  | 0 |  | 2 (20%) |  | 0 |  | 0 |  | 0 |  |  | 1 (9%) |  |
| Obstetrics and gynaecology doctor |  | 3 (27%) |  | 2 (18%) |  | 1 (10%) |  | 2 (12%) |  | 1 (10%) |  | 5 (29%) |  |  | 4 (36%) |  |
| Anaesthetist |  | 0 |  | 0 |  | 0 |  | 0 |  | 0 |  | 0 |  |  | 0 |  |
| General practitioner |  | 1 (9%) |  | 1 (9%) |  | 0 |  | 0 |  | 0 |  | 1 (6%) |  |  | 0 |  |
| Operating department practitioner |  | 0 |  | 0 |  | 0 |  | 0 |  | 0 |  | 0 |  |  | 0 |  |
| Physiotherapist |  | 0 |  | 0 |  | 0 |  | 0 |  | 0 |  | 0 |  |  | 0 |  |
| Member of an interested organisation of charity |  | 0 |  | 1 (9%) |  | 1 (10%) |  | 2 (12%) |  | 1 (10%) |  | 0 |  |  | 0 |  |
| Medico-legal professional |  | 0 |  | 0 |  | 0 |  | 0 |  | 0 |  | 0 |  |  | 0 |  |
| Researcher |  | 0 |  | 1 (9%) |  | 0 |  | 1 (6%) |  | 1 (10%) |  | 0 |  |  | 0 |  |
| Other |  | 0 |  | 2 (18%) |  | 0 |  | 3 (18%) |  | 0 |  | 2 (12%) |  |  | 0 |  |


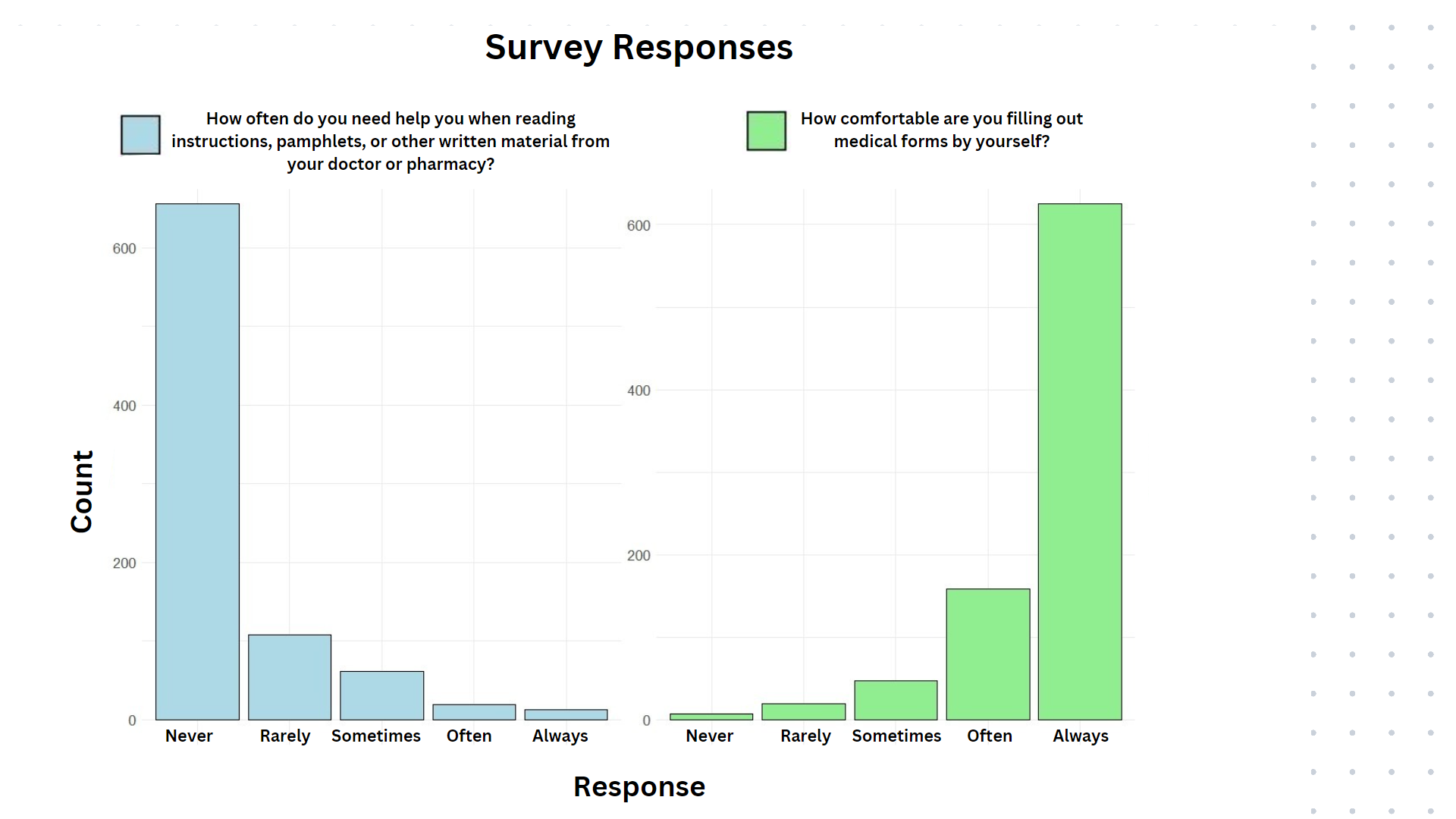


Supplementary figure 3: Bar graph of responses for the two health literacy questions

Supplementary table 3: Medians and interquartile ranges for the surprise and ease of understanding questions.

|  |  | 10 Dots | 10 People | 100 Dots | 100 People | Bar chart | Pie chart | Words |
| --- | --- | --- | --- | --- | --- | --- | --- | --- |
| 99.5/100 | Surprise question | Median 1 IQR 1-2 | Median 1  IQR 1-2 | Median 1  IQR 1-2 | Median 1  IQR 1-1.25 | Median 1  IQR 1-2 | Median 1  IQR 1-2 | Median 1  IQR 1-2 |
|  | Ease of understanding | Median 9  IQR 8-10 | Median 9.5  IQR 8-10 | Median 9  IQR 8-10 | Median 9  IQR 8-10 | Median 9  IQR 8-10 | Median 9  IQR 8-10 | Median 9  IQR 8-10 |
| 2/100 | Surprise question | Median 5  IQR: 7.75-9 | Median 9  IQR 8-9 | Median 9  IQR 8-9 | Median 8  IQR 8-9 | Median 9  IQR 7-9 | Median 8  IQR 7-9 | Median 8  IQR 7-9 |
|  | Ease of understanding | Median 9  IQR 7-10 | Median 9  IQR 7-10 | Median 9  IQR 8-10 | Median 10  IQR 9-10 | Median 9  IQR 8-10 | Median 9  IQR 8-10 | Median 10  IQR 8-10 |
| 50/100 | Surprise question | Median 5  IQR 5-6 | Median 5  IQR 4-5 | Median 5  IQR 4-5 | Median 5  IQR 4-5 | Median 5  IQR 3.75-5 | Median 5  IQR 3-5 | Median 5  IQR 4-6 |
|  | Ease of understanding | Median 9  IQR 8-10 | Median 9  IQR 8-10 | Median 9  IQR 8-10 | Median 10  IQR 8-10 | Median 10  IQR 8-10 | Median 10  IQR 8-10 | Median 9  IQR 8-10 |
| 0.4/100 | Surprise question | Median 10  IQR 9-10 | Median 10  IQR 9-10 | Median 10  IQR 9-10 | Median 10  IQR 9-10 | Median 10  IQR 8-10 | Median 9  IQR 8-10 | Median 10  IQR 8-10 |
|  | Ease of understanding | Median 9  IQR 6-10 | Median 8  IQR 6-10 | Median 9  IQR 7-10 | Median 9  IQR 7-10 | Median 9  IQR 7-10 | Median 9  IQR 8-10 | Median 8  IQR 4-9 |

Supplementary table 4: Scores for the ‘Biggest risk’ questions.

| Biggest risk | | 10 circles | 10 people: | 100 circles: | 100 people: | Bar chart: | Pie chart: | Words |
| --- | --- | --- | --- | --- | --- | --- | --- | --- |
| 2/100 vs 4/100 | Correct | 117/120 (97.5%) | 105/124 (84.7%) | 130/136 (95.6%) | 127/129  (98.5%) | 112/120  (93.3%) | 93 /101  (92.1%) | 123/128 (96.1%) |
|  | Incorrect | 3/120  (2.5%) | 19/124 (15.32%) | 5/136  (3.7%) | 2/100  (1.55%) | 8/120 (6.67%) | 8/101  (7.92%) | 5/128 (3.90%) |
|  | No response |  |  | 1/136 (0.74%) |  |  |  |  |
| 96/100 vs 98/100 | Correct | 116/120 (96.67%) | 116/124 (93.55%) | 129/136  (94.85%) | 127/129  (98.45%) | 114/120 (95%) | 94/101  (93.1%) | 125/128 (97.7%) |
|  | Incorrect | 3/120  (2.5%) | 8/124  (6.45%) | 6/136 (4.41%) | 2/129 (1.55%) | 6/120  (5%) | 7/101 (6.93%) | 3/128 (2.34%) |
|  | No response | 1/120  (0.83%) |  | 1/136 (0.74%) |  |  |  |  |
| 2/100 vs 0.4/100 | Correct | 112/120 (93.3%) | 119/124 (95.97%) | 129/136 (94.85%) | 125/129 (96.9%) | 114/120  (95%) | 97/101 (96.03%) | 117/128  (91.41%) |
|  | Incorrect | 7/120 (5.47%) | 4/124 (3.23%) | 6/136  (4.41%) | 4/129 (3.10%) | 6/120  (5%) | 3/101 (2.97%) | 11/128 (8.6%) |
|  | No response | 1/120 (0.83%) | 1/124 (0.83%) | 1/136  (0.74%) |  |  | 1/101 (0.99%) |  |
| 99.5/100 vs 0.4/100 | Correct | 115/120 (95.83%) | 122/124 (98.39%) | 127/136  (93.4%) | 124/129  (96.12%) | 114/120 (95%) | 98/101 (97.03%) | 120/128 (93.75%) |
|  | Incorrect | 4/120 (3.33) | 2/124 (1.61%) | 9/136 (6.62%) | 3/129 (2.33%) | 6/120  (5%) | 3/101 (2.97%) | 8/128 (6.25%) |
|  | No response | 1/120 (0.83%) |  |  | 2/129 (1.55%) |  |  |  |


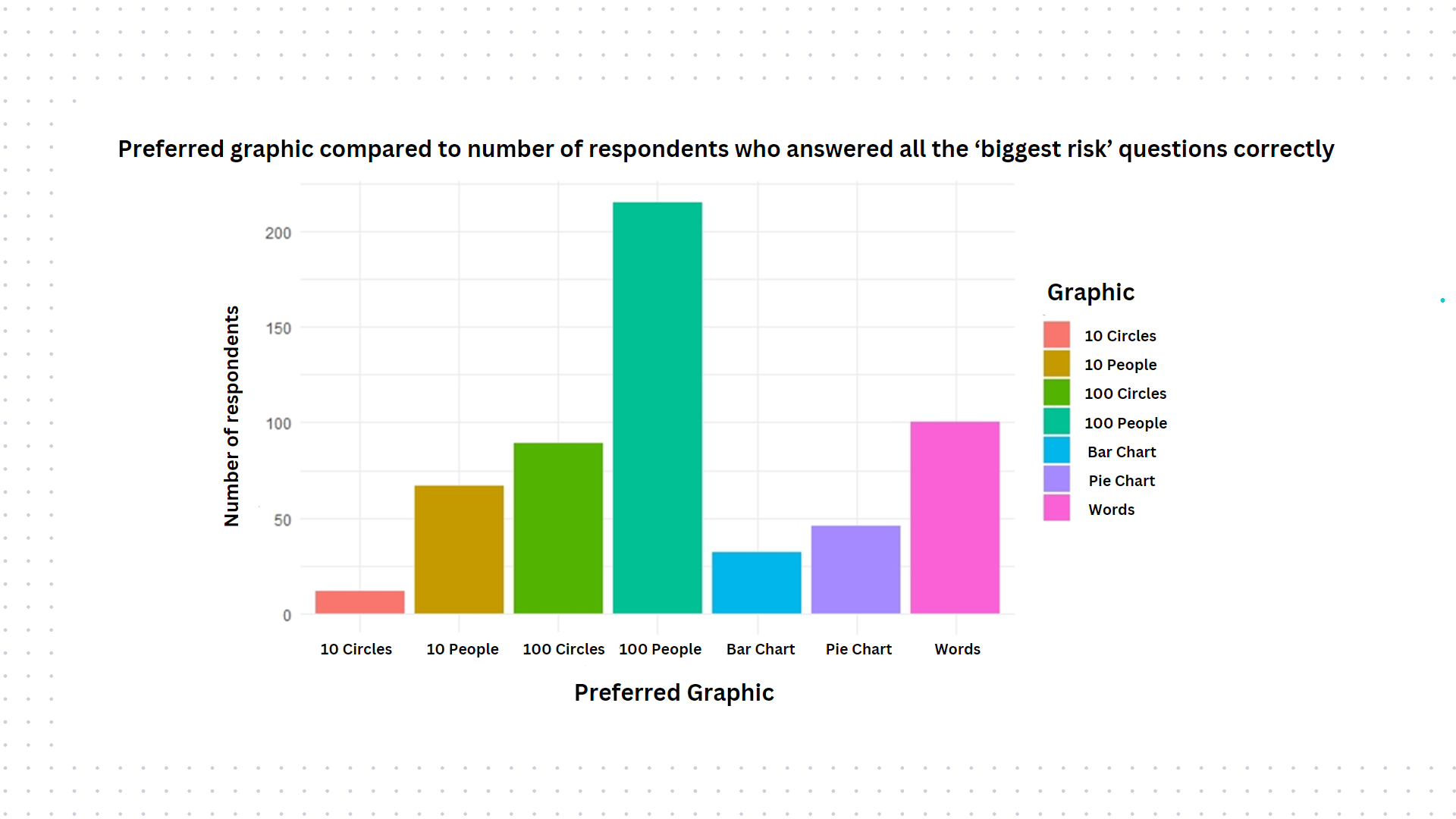


Supplementary figure 4: Preference of graphic compared the performance when identifying the largest of two risks.


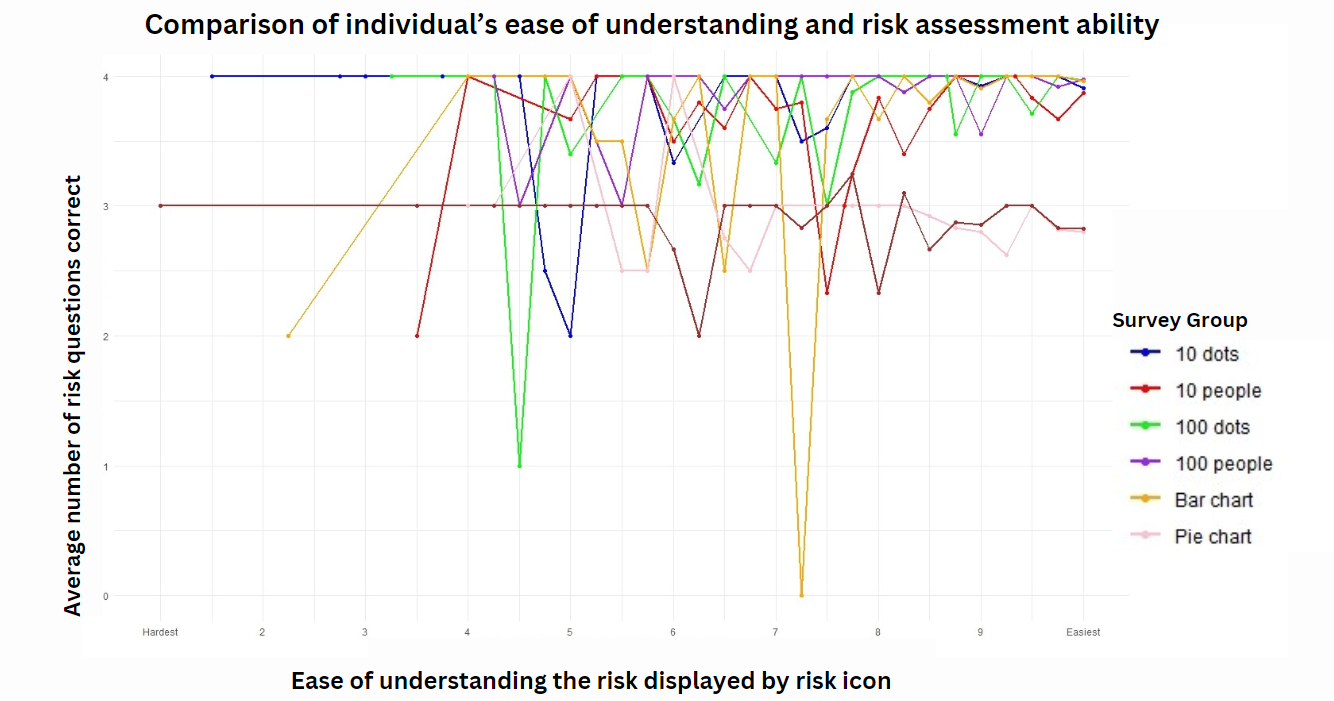


Supplementary figure 5: Individual’s ease of understanding compared to their ability to assess risk according to the graphic type they received.


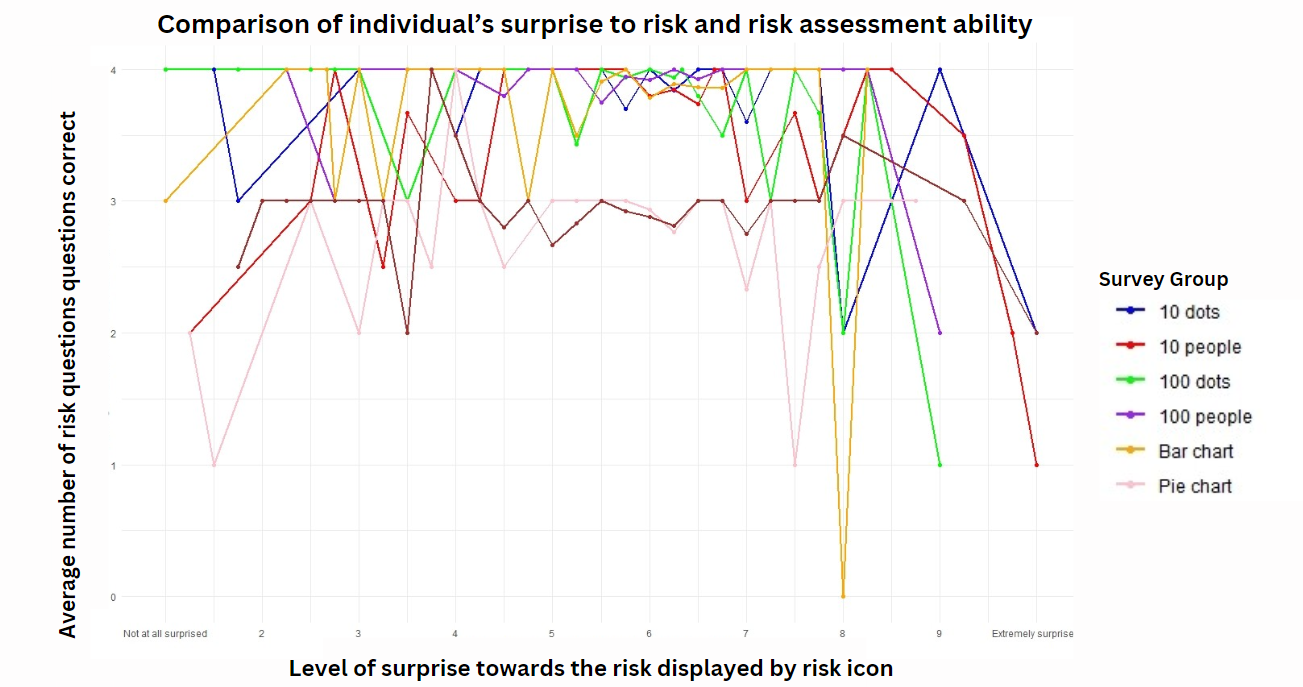


Supplementary figure 6: Individual’s surprise compared to their ability to assess risk according to the graphic type they received.


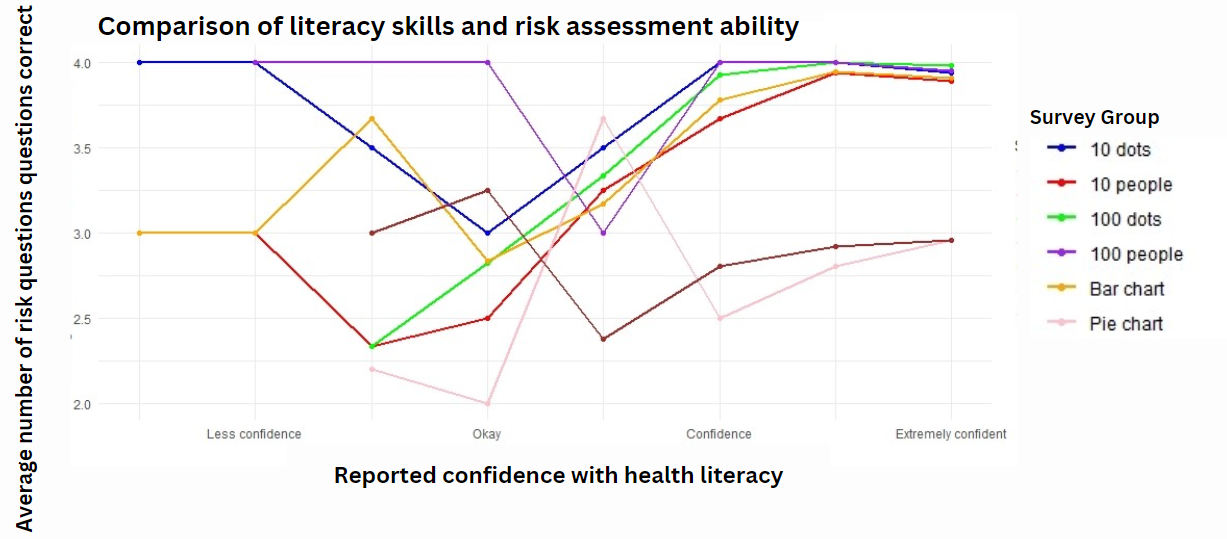


Supplementary figure 7: Individual’s literacy ability compared to their risk assessment ability.
